# Assessing the potential of solubility trapping in unconfined aquifers for subsurface carbon storage

**DOI:** 10.1038/s41598-022-24623-6

**Published:** 2022-11-28

**Authors:** Mouadh Addassi, Abdirizak Omar, Hussein Hoteit, Abdulkader M. Afifi, Serguey Arkadakskiy, Zeyad T. Ahmed, Noushad Kunnummal, Sigurdur R. Gislason, Eric H. Oelkers

**Affiliations:** 1 King Abdullah University of Science and Technology (KAUST), Thuwal, 23955-6900 Saudi Arabia; 2 Environmental Protection, Saudi Arabian Oil Company, Dhahran, Saudi Arabia; 3 Institute of Earth Sciences, University of Iceland, Sturlugötur 7, 102 Reykjavík, Iceland; 4 Géosciences Environnement Toulouse (GET), CNRS UMR 5563, Toulouse, France

**Keywords:** Carbon capture and storage, Environmental impact

## Abstract

Carbon capture and storage projects need to be greatly accelerated to attenuate the rate and degree of global warming. Due to the large volume of carbon that will need to be stored, it is likely that the bulk of this storage will be in the subsurface via geologic storage. To be effective, subsurface carbon storage needs to limit the potential for CO_2_ leakage from the reservoir to a minimum. Water-dissolved CO_2_ injection can aid in this goal. Water-dissolved CO_2_ tends to be denser than CO_2_-free water, and its injection leads immediate solubility storage in the subsurface. To assess the feasibility and limits of water-dissolved CO_2_ injection coupled to subsurface solubility storage, a suite of geochemical modeling calculations based on the TOUGHREACT computer code were performed. The modelled system used in the calculations assumed the injection of 100,000 metric tons of water-dissolved CO_2_ annually for 100 years into a hydrostatically pressured unreactive porous rock, located at 800 to 2000 m below the surface without the presence of a caprock. This system is representative of an unconfined sedimentary aquifer. Most calculated scenarios suggest that the injection of CO_2_ charged water leads to the secure storage of injected CO_2_ so long as the water to CO_2_ ratio is no less than ~ 24 to 1. The identified exception is when the salinity of the original formation water substantially exceeds the salinity of the CO_2_-charged injection water. The results of this study indicate that unconfined aquifers, a generally overlooked potential carbon storage host, could provide for the subsurface storage of substantial quantities of CO_2_.

## Introduction

Carbon capture and storage (CCS) is one of the key strategies available to combat the rising levels of greenhouse gases in the atmosphere. Some estimates suggest that global CCS efforts must successfully store in excess of several gigatons annually to limit global warming to no more than 1.5 or 2 °C above pre-industrial levels^1^. Current CCS efforts, however, store less than ~ 50 million tons of CO_2_ annually. It is, therefore, critical to accelerate the adoption of carbon storage solutions^2–5^.

Numerous subsurface CO_2_ storage methods have been advocated and studied over the past few decades to enable large-scale storage. Some of the targeted geologic media, such as some deep confined saline aquifers and depleted oil and gas reservoirs, have robust caprocks capable of maintaining buoyant supercritical CO_2_ in the subsurface for extended time-frames^6–9^. Other potential subsurface carbon storage formations, such as fractured reactive basalts or unconfined saline aquifers, may not contain caprocks and have fluid pathways that would enable buoyant single-phase CO_2_ to rapidly return to the surface^8^. Injection of carbonated water has been proposed as a solution to this issue^10–14^. This method introduces an already stable and non-buoyant phase into the reservoir, which allows the storage of CO_2_ directly by solubility trapping^14–18^. Moreover, water-dissolved CO_2_ can also promote the dissolution of divalent metal-bearing silicate minerals leading to the formation of carbonate minerals fixing the injected dissolved gas in the solid-state^17,19–22^.

The injection of CO_2_-charged water into the subsurface overcomes the slow dissolution of CO_2_ into reservoir fluids if injected as a single phase. Although carbon dioxide dissolution into water is fast, and equilibrium is generally assumed for this reaction at the CO_2_-water interface^23–25^, in the subsurface the overall dissolution process is slow. Carbon dioxide dissolution in subsurface rock formations is limited by CO_2_ diffusion into the water at the CO_2_-water interface and the subsequent CO_2_-charged water convection driven by density contrasts between the CO_2_-saturated and unsaturated brines^26^. The slow reservoir scale dissolution of CO_2_ is a bottleneck in exploiting solubility trapping as a primary storage method in geologic formations.

The goal of this study is to assess the potential of CO_2_ storage in unconfined aquifers though the injection of carbonated water through a series of comprehensive chemical transport calculations. Particular attention is focused on the effect of pressure, temperature, and salinity gradients on the stability of carbonated water injected into the subsurface. The purpose of this manuscript is to report the results of this computational study and to use these results to assess the potential for subsurface carbon storage in unreactive unconfined aquifers.

## Methods

The currently study considers only reactions among the fluid phases in the system; mineral dissolution and precipitation reactions have been excluded for several reasons. First, with the exception of relatively rapidly reacting minerals such as evaporites, carbonates and some silicates such as basaltic glass and olivine, the dissolution rates of many minerals, including those of clays and quartz, which dominate many sedimentary rocks are too slow to affect greatly model results. Secondly, the inclusion of mineral-fluid reactions would require the choice of host-rock mineralogy making computed results less general.

TOUGHREACT version 3.32^27^ was used to calculate the multiphase flow of CO_2_, including CO_2_ solubility and the transport in aqueous fluids. The results of solubility calculations from this code, which are critical for this study, were validated by comparison with corresponding solubilities calculated using CMG-GEM version 2018.10^28^, and with available experimental data. A close correspondence was found between all calculated and experimental values.

### Transport model

The governing equations for multiphase multicomponent transport in porous media are given by the material/mass balance equations, Darcy’s law, and the thermodynamic equilibrium between the phases^29,30^. The general form of these equations is described in this section, highlighting relations relevant to this work. The mass of each component in the phases present in the system can be described by material balance given by:

1 $$\frac{{\partial M_{\kappa } }}{\partial t}\, = \, - \nabla F_{\kappa } + q_{\kappa } \quad \kappa \,\, = \,1, \ldots ,n_{c} ,$$

where $M$ represents the mass accumulation, $F$ refers to the mass flux, and $q$ denotes a source or sink term. The components, e.g., water and CO_2_ are represented by the index $\kappa$ for each of the $n_{c}$ components in the system.

The mass accumulation term $M_{\kappa }$ is given by:

2 $$M_{\kappa } = \phi \sum\limits_{\beta } {S_{\beta } \rho_{\beta } X_{\kappa ,\beta } } ,$$

where $\phi$ denotes the porosity,$S_{\beta }$ refers to the saturation,$\beta$ represents the phase i.e., gas or aqueous fluid, $\rho_{\beta }$ refers to the mass density, and $X_{\kappa ,\beta }$ represents the mass fraction of component $\kappa$ in phase $\beta$.

The flux of the $\kappa$*th* component, $F_{\kappa }$, is written as:

3 $$F_{\kappa } = \sum\limits_{\beta } {X_{\kappa ,\beta } \rho_{\beta } {\mathbf{u}}_{\beta } } ,$$

where ${\mathbf{u}}_{\beta }$ denotes the phase velocity, which is described by Darcy’s law^31^ as:

4 $${\mathbf{u}}_{\beta } = - \frac{{{\mathbf{k}}k_{r,\beta } }}{{\mu_{\beta } }}(\nabla p_{\beta } - \rho_{\beta } {\mathbf{g}}),$$

where ${\mathbf{k}}$ signifies the absolute permeability of the porous medium, and $k_{r,\beta }$, $\mu_{\beta }$, and $\rho_{\beta }$ represent the relative permeability, viscosity, and mass density, respectively, of the phase $\beta$. The symbol $p$ denotes the pressure and ${\mathbf{g}}$ the gravitational acceleration.

The diffusive flux, ${\mathbf{J}}$, is derived from Fick’s law^32^:

5 $${\mathbf{J}}_{\beta } = - c_{\beta } {\mathbf{D}}_{\beta } \nabla x_{\beta } ,$$

where ${\mathbf{D}}_{\beta }$ corresponds to the diffusion coefficient in phase $\beta$.

Local thermodynamic equilibrium is expressed as the equality of the fugacities of each component in the phases $\beta_{1}$ and $\beta_{2}$:

6 $$f_{{i,\beta_{1} }} (T,p,x_{{j,\beta_{1} }} ) = f_{{i,\beta_{2} }} (T,p,x_{{j,\beta_{2} }} ), \quad i = 1,...,n_{c} {, } \quad j = 1,...,n_{c} - 1,$$

where $f_{i,\beta }$ stands for the fugacity of a component $i$ in phase $\beta$. The fugacity can be determined using an equation of state. The equations presented above are implemented in TOUGHREACT^33,34^ and control multicomponent transport in the physical domains represented in the model calculations presented below.

#### Carbon-dioxide solubility

The interaction between CO_2_ and water is an important phenomenon during carbon storage. Solubility trapping, a key sequestration mechanism, is driven by this interaction^15,23,35,36^. The dissolution of gaseous CO_2_ in water is a relatively rapid reaction at the local scale, and therefore local thermodynamic equilibrium is assumed between the gaseous CO_2_ and aqueous phases. This local equilibrium is expressed in terms of the fugacities in each phase, such that:

7 $$f_{i,g} = f_{i,aq} {, } \quad i = 1,...,n_{c} ,$$

where, $f_{i,g}$ represents the fugacity of the $i{\text{th}}$ component in the gas phase, and $f_{i,aq}$ represents the fugacity of the $i{\text{th}}$ component in the aqueous phase. The fugacity of CO_2_ in the gas phase is related to its partial pressure by:

8 $$f_{{CO_{2} ,g}} = \varphi P_{{CO_{2} }} ,$$

where $\varphi$ denotes the fugacity coefficient estimated using the Peng-Robinson EOS^37^, and $P_{{CO_{2} }}$ refers to the partial pressure of CO_2_. In the aqueous phase, the fugacity of CO_2_ is calculated.

using the extended Henry’s law^38^:

9 $$f_{{CO_{2} ,aq}} = y_{{CO_{2} }} H_{{CO_{2} }} ,$$

where, $y_{{CO_{2} }}$ stands for the mole fraction of CO_2_ in the aqueous phase and $H_{{CO_{2} }}$ represents Henry’s constant. In pure water, Henry’s constant, which is a function of pressure, temperature, the universal gas constant, and the partial molar volume with respect to a reference pressure, is given by:

10 $$\ln H_{{CO_{2} }} = \ln H_{{_{{CO_{2} }} }}^{*} + \frac{{\overline{v}_{i} (p - p^{*} )}}{RT}.$$

In saline water, a correction is made to Henry’s constant to account for salinity, such that:

11 $$\ln \left( {\frac{{H_{{brine,CO_{2} }} }}{{H_{{CO_{2} }} }}} \right) = k_{{s,CO_{2} }} m_{s} ,$$

where $H_{{brine,CO_{2} }}$ denotes Henry’s constant for CO_2_ in the brine, $H_{{CO_{2} }}$ corresponds to Henry’s constant for CO_2_ in pure water, $k_{{s,CO_{2} }}$ designates the Setchenov salting-out coefficient for a CO_2_-water aqueous phase, and $m_{s}$ refers to the molality of the dissolved salt.

Carbon-dioxide solubility in TOUGHREACT and CMG-GEM is calculated following Henry’s law. In TOUGHREACT, the effect of salinity, temperature, and pressure on the fugacity coefficients of CO_2_ is calculated within the ECO2N module using the H_2_O-CO_2_ mutual solubility model of Spycher and Pruess^39–41^. In CMG-GEM, the effects of salinity, temperature, and pressure on Henry’s constants are accounted for by either Harvey’s correlation^42^ or the Li and Nghiem correlation^43^.

## Numerical examples

This study aims to assess the limits of solubility trapping in unconfined aquifers by the injection of CO_2_-charged water. Towards this goal, several numerical examples have been conducted. The results of each example are compared to a reference case to illustrate the effects of varied parameters. As a reference case, a simplified reservoir with a hydrostatic pressure gradient represented by a radial model is considered. In this reference case, carbonated water is continuously injected for 100 years. The distribution of CO_2_ during injection and post injection is computed over and beyond this time period.

**Figure 1:**
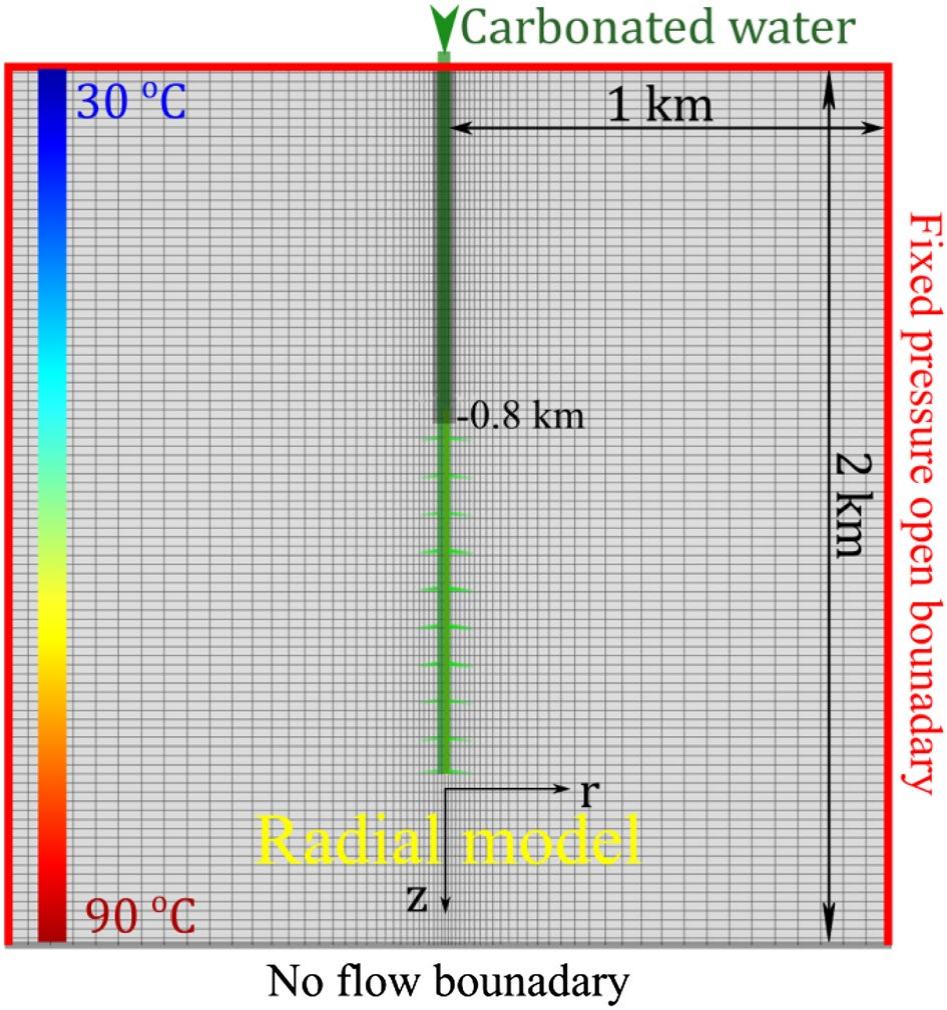
A schematic of the radial simulation domain considered in this study, including boundary conditions, injection zone, and the temperature gradient for the reference system. The plot background shows the numerical domain with 20 m blocks in the z direction and varying grid size in the radial direction. The finest grid blocks in the radial direction are 5 m, near the well, increasing to 50 m further away from the well.

A single system geometry was selected to illustrate the effect of varying parameters on water-charged CO_2_ injection efforts. The choice of a single system geometry allows for direct comparison of the model results. A schematic illustration of the modelled subsurface system is shown in Fig. 1. The domain consists of a radial model with a 1 km radius and 2 km total depth. The injection interval is 800 m long, starting from a depth of 800 m and extending down to a depth of 1600 m. The thickness of the injection zone was set to minimize pressure buildup. It might be uncommon to find continuous reservoir rock without heterogeneities in a vertical interval. However, this could be thought of as a well penetrating multiple formations with a net pay thickness of 800 m, which may include non-reservoir streaks. It should be possible to have a ‘large’ injection zone in unconfined aquifers, as we are not targeting a specific zone below a seal. This contrasts with conventional reservoirs where the injection zone is limited to the targeted traps. An example of that is when using commingle wells to access stacked reservoirs, including geothermal reservoirs where the perforated intervals are commonly within 1000-2000m^44,45^. A no-flow boundary is located at a depth of 2 km. This represents a non-permeable formation. A fixed-pressure open flow boundary is located at the top and the edges of the radial model.

The total pore volume in the system is approximately $6 \times 10^{8}$ m^3^_._ A rough estimate of the maximum solubility storage capacity of this system is calculated to be 12 million metric tons of CO_2_, assuming the average CO_2_ solubility in the reservoir aqueous phase is 4% by mass, and that half of the pore space is available for storage. This mass of CO_2_ storage volume would allow an injection rate of 100,000 metric tons/year for 120 years to fill the domain completely with CO_2_ saturated water. Such an injection would require a total injection of 2.5 to 3.5 million metric tons of fluid into the subsurface over 120 years. This injection rate is within the range of geothermal wells. Average water re-injection rates from geothermal sites around the world range from 0.2 to 5 Mt/year^46–48^, see Fig. S1. The injected water is assumed to enter the reservoir with a temperature of 40 °C. The specific heat and the wet heat conductivity of the rock are assumed to be 1000 J/(kg·K) and 2.0 W/(m·K), respectively. These values are within the range of reported values for different rock types, including sedimentary and volcanic rock^49^. A linear function is assumed for the relative permeability with a critical gas saturation of 0.1.

### Model parameters

Numerous parameters have been varied to illuminate their effects on the fate of injected water dissolved CO_2_. Table 1 summarizes the limits of the parameters considered in this study. The main parameters varied include the temperature and its gradient, the salinity and its gradient, the vertical to horizontal permeability ratio and the perforation thickness of the dissolved gas injection.

**Table 1:** Model parameters and their impact on solubility storage potential.

	Temperature gradient	Salinity	Salinity gradient	Injection water		Injection zone		Permeability		W\CO_2_ ratio*
				Temperature	Salinity	Top	Bottom	k_v_	K_h_	
	°C/km	ppm	ppm/m	°C	ppm	m	m	md	md	kg/kg
Reference case	30	35,000	0	40	35,000	800	1600	100	100	25
Temperature gradient	**18**	35,000	0	40	35,000	800	1600	100	100	25
	**50**	35,000	0	40	35,000	800	1600	100	100	26
Ignoring thermal effects	30	35,000	0	**Same as reservoir**	35,000	800	1600	100	100	28
Constant reservoir salinity	30	**10,000**	0	40	35,000	800	1600	100	100	25
	30	**100,000**	0	40	35,000	800	1600	100	100	35*
Injecting reservoir fluid	30	**10,000**	0	40	**10,000**	800	1600	100	100	22
	30	**100,000**	0	40	**100,000**	800	1600	100	100	35
Salinity gradient	30	–	**10**	40	35,000	800	1600	100	100	25
	30	–	**100**	40	35,000	800	1600	100	100	25
Perforation zone thickness	30	35,000	0	40	35,000	800	**1200**	100	100	26
	30	35,000	0	40	35,000	800	**2000**	100	100	25
Injection depth	30	35,000	0	40	35,000	**1000**	**1800**	100	100	23
	30	35,000	0	40	35,000	**1700**	**2000**	100	100	**19**
Permeability ratio	30	35,000	0	40	35,000	800	1600	**10**	100	20
	30	35,000	0	40	35,000	800	1600	**200**	100	27

Significant values are in bold.

*The water to CO_2_ ratio was fixed to be the minimum value without allowing CO_2_ to escape to the surface within 100 years of carbonated water while injecting 100,000 metric tons CO_2_/year.

## Results

### The solubility of CO_2_ and the density of CO_2_-charged water as a function of depth

**Figure 2:**
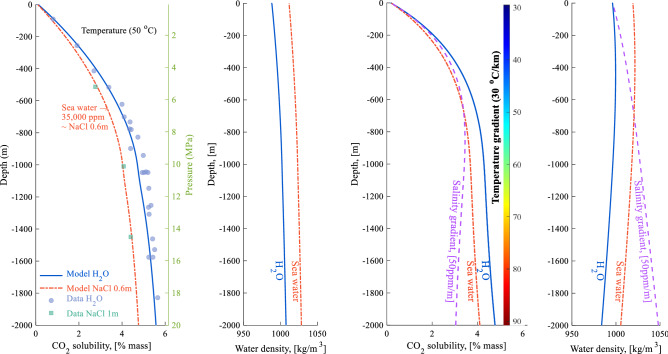
The left pair of plots show CO_2_ solubility and density of CO_2_-saturated water as a function of depth. The temperature in the system is constant at 50 °C and two salinity cases are shown (0 ppm—pure water; and 35,000 ppm—seawater). The experimental data shown for CO_2_ solubility was compiled by (circle filled with blue colour)Appelo et al.^50^ and (square filled with green colour) Koschel et al.^51^. The right pair of plots show CO_2_ solubility and CO_2_-saturated water density as a function of depth, assuming a temperature gradient of 30 °C/km. Three salinity cases are shown; pure water, seawater, and a vertical salinity gradient of 50 ppm/m. The model results were calculated using ECO2N EOS in TOUGHREACT. A normal hydrostatic pressure gradient was assumed for all cases, as shown on the secondary y-axis of the left-most plots.

The solubility of CO_2_ in water, and the density of CO_2_-charged water as a function of depth depend on many factors including the gas pressure, temperature, and salinity. Figure 2 shows examples of how these properties vary versus depth, assuming a hydrostatic pressure gradient. At a constant temperature of 50 °C, the solubility and density of carbonated water increase with increasing depth. The CO_2_ solubility and density of pure water and saline seawater differ somewhat. Increasing salinity lowers CO_2_ solubility but increases the density of the water.

The calculated CO_2_ solubility values from the model calculations are in close agreement with experimental data compiled by Appelo et al.^50^ and Koschel et al.^51^. The thermodynamic properties of CO_2_/brine mixtures calculated using TOUGHREACT, and GEM were compared to experimental data in^52^. Both numerical codes captured the solubility trends well, and good matches were obtained against experimental data for different ranges of temperature, pressure, and salinity investigated.

The presence of a temperature or salinity gradient, however, changes significantly the variation of CO_2_ solubility and the density of CO_2_ saturated water with depth. A temperature gradient of 30 ^◦^C/km results in lower CO_2_ solubility with depth compared to the constant temperature case as shown in Fig. 2. The solubility of CO_2_ in water increases with increasing pressure and decreases with increasing temperature and salinity. Down to a depth of approximately 600–800 m, the pressure effects dominate, resulting in an increase of CO_2_ solubility with depth (for a temperature gradient of 30 ^◦^C/km). Below approximately 600–800 m depth, the solubility curve flattens for pure water and seawater, and it reverses in the presence of a 50 ppm/m salinity gradient. The carbonated water density curve, thereby, shows how the temperature gradient results in reduced water density with depth after a few hundred meters of depth. The effect of fluid density decreasing due to increasing temperature dominates the density increase due to increasing pressure with depth for both pure water and seawater. The presence of a 50 ppm/m salinity gradient in the reservoir, combined with the effect of pressure and solubility, overrides the effect of the temperature gradient on water density, and results in a net increase of water density with increasing depth.

### The fate of injected CO_2_ into a homogeneous hydrostatically pressured reservoir

The first model calculation focuses on a system having a constant porosity of 0.1 and constant permeability of 100 mD in the vertical and horizontal directions. Although it is unrealistic to encounter a homogenous reservoir with constant porosity and permeability, the selected values are within the lower range for sandstone^53^, as well as other rock types, including basaltic rocks^54^. The impact of the ratio between vertical and horizontal permeability are investigated later in this study. The surface temperature is taken as 30 ^◦^C and temperature gradient as 30 °C/km. The salinity of the reservoir and the injected water is assumed constant and equal to a 35,000 ppm NaCl solution, which is approximately equal to that of seawater.

**Figure 3:**
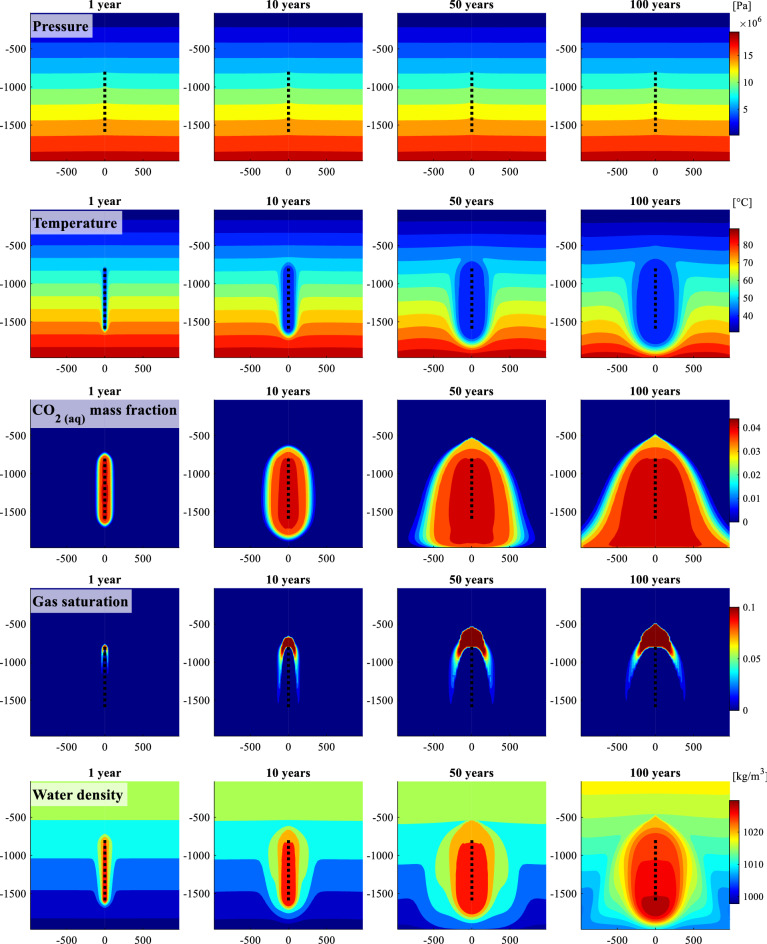
Simulation results for the reference case considered in this study. 2D cross-sections of the (from top to bottom) pressure, temperature, CO_2_ mass fraction in the liquid, gas saturation, which is the mass fraction of exsolved CO_2_ in the pore fluids, and water density for the reference system after 1, 10, 50 and 100 years of carbonated water injection. Color bars indicating the range for each of the properties are displayed on right side of the plots. The dotted line marks the gas-charged water injection zone. This homogeneous hydrostatically pressured reference system assumes a 30 °C/km gradient, constant salinity of 35000 ppm (seawater), and water injection temperature of 40 °C. The water to CO_2_ injection mass ratio is 25:1.

Figure 3 illustrates 2D cross-section time snapshots for the pressure, temperature, CO_2_ mass fraction in the liquid, gas saturation, and water density. Note that gas saturation in this study represents the fraction of exsolved CO_2_ mass in the pore fluids. The fluid pressure profile remains fairly constant as the injection rate is kept constant. The pressure buildup in these systems was insignificant due to the large injection zone, open-flow boundary conditions, and the assumed porosity and permeability conditions. The temperature near the well decreases over time due to cooling by the injected carbonated water, which improves the storage capacity during injection. The gas saturation profiles in Fig. 3 show that part of the CO_2_ exsolves at the top of the region containing saturated carbonated water, then redissolves as it moves into the non-CO_2_ saturated zones higher up in the system.

Furthermore, we modeled the fate of the injected CO_2_ post-injection. We monitored the fluid flow for 100 years after the 100-year injection period, as presented in Fig. S2. After the end of the 100-year injection, the carbonated water sinks towards the bottom of the system then is transported away due to the open boundary conditions. This result is consistent with those of^55^.

The water to CO_2_ mass injection ratio for this homogeneous hydrostatically pressured reference system is 25:1. This ratio was the maximum injection ratio of this system, where the exsolved CO_2_ zone is stable and does not reach the surface. Lowering the water to CO_2_ ratio to 24:1 results in some of the exsolved CO_2_ reaching the surface after approximately 40 years of injection (see Fig. S3).

### Impact of temperature gradient on the fate of injected gas-charged CO_2_

**Figure 4:**
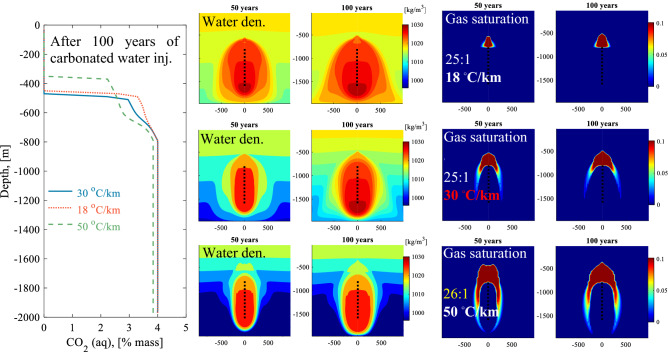
Left plot: A comparison of CO_2_ concentration profiles at the well location (r = 0) as a function of depth after 100 years of injection for three temperature gradients of 18, 30 (reference case) and 50 °C/km. The water to CO_2_ injection mass ratio is 25:1 for the 18, and 30 °C/km gradient, and 26:1 for the 50 °C/km gradient. Center and right plots: 2D cross-sections for the water density and the gas saturation, which is the mass fraction of exsolved CO_2_ in the pore fluids, for the three temperature gradients after 50 and 100 years of carbonated water injection.

The impact of the temperature gradient of CO_2_ solubility storage was explored by considering gradients of 18, 30 (reference system), and 50 °C/km. Figs. S5,S6 illustrate 2D cross-section time snapshots for the temperature, CO_2_ mass fraction in the liquid, gas saturation, and water density for the 30 and 50 °C/km gradient. Figure 4 compares the calculated dissolved CO_2_ concentration versus depth profiles at the well location (r = 0) for the three temperature gradients. Both the 18 and 30 °C/km gradient cases have the same injected water to CO_2_ ratio of 25:1. A less steep temperature gradient (18 °C/km) should improve the storage capacity in the reservoir as CO_2_ is more soluble at lower temperatures. However, Fig. 4 shows the top of the CO_2_ concentration profiles versus depth is slightly deeper for 30 °C/km compared to the 18 ^◦^C/km case, leading to marginally better storage security for the 30 °C/km case. This counter-intuitive result stems from differing fluid densities; the carbonated water tends to sink more rapidly in the lower temperature gradient system. The contrast between the density of the injected carbonated water and the initial water in place increases with increasing temperature gradient, which accelerates the flow of carbonated water downwards. This behavior results in a more concentrated zone of carbonated water near the injection well.

For the lowest temperature gradient of 18 °C/km, the exsolved gas is only seen at the top of the injection zone, as shown in Fig. 4. The exsolved gas zone expands deeper around the injection zone with an increasing temperature gradient. The increasing density gradient between the injected carbonated water and the formation fluids due to increasing temperature gradients, pushes the injected water to travel more downwards than upwards or vertically. Further, the CO_2_ solubility decreases with increasing temperature contributing to a solubility difference between the zone cooled by the injected water and the surrounding reservoir water. This leads to increased CO_2_ exsolution. In fact, the 50 ^◦^C/km gradient case required a slightly higher water to CO_2_ ratio of 26:1 to avoid the leakage of exsolved CO_2_ at the surface.

### Impact of subsurface temperature changes due to fluid injection

In general, if the injected water is colder than the reservoir water, it results in a positive impact on CO_2_ solubility in the subsurface system due to the retrograde solubility of CO_2_. A number of past studies have assumed that the temperature of the injected water has no significant effect on the subsurface fluid temperatures, and thus the thermal effects were ignored^12,56^. The model simulations performed in this study indicate that the injection of fluid into a porous reservoir with a temperature gradient of 30 ^◦^C/km or above will significantly alter the temperature near the injection well, as shown in Fig. 3. A comparative calculation for a system neglecting the heating or cooling effect of injected fluids was run to assess the impact of this assumption on computed results. The results of this calculation are shown in Fig. S4. In the absence of formation fluid heating or cooling due to fluid injection, the minimum water to CO_2_ mass ratio that can be injected while avoiding exsolved CO_2_ escaping to the surface increases to 28:1. Thus, the secure injection of CO_2_ into this system requires 12% more water per unit mass of CO_2_ injected compared to the reference system, which has a thermal gradient of 30 ^◦^C/km. In the case of injecting CO_2_-charged water into a system having an 18 ^◦^C/km temperature gradient, the average temperature in the injection zone is ~ 40 °C. Thus, the isothermal system behaves similarly to that of the 18 ^◦^C/km temperature gradient system^56^. For a temperature gradient of 30 °C/km or above, however, ignoring thermal effects would lead to an overestimation of the water needed to avoid CO_2_ leakage to the surface.

### Impact of formation and injection water salinity

The effect of salinity on water-dissolved CO_2_ injection was studied by varying the initial salinity of the formation water from 35,000 ppm for the reference system, to either 10,000 or 100,000 ppm. Two cases are considered in each of these systems. In the first, it is assumed that the injected water has the same salinity as the reference system, which is 35,000 ppm. In the second, it is assumed that the injected water has the same salinity as the reservoir fluids.

**Figure 5:**
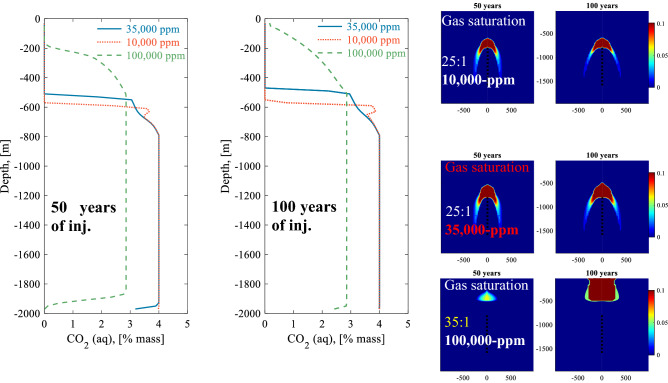
Left and center plots: Comparison of CO_2_ concentration profiles at the well versus depth after 50 and 100 years of injection for the injection of CO_2_ charged water having a seawater salinity with into reservoirs having an initial salinity of 10,000, 35,000 (the reference case), and 100,000 ppm. The injection water to CO_2_ mass ratio is 25:1 for the 10,000 and 35,000, ppm initial salinities, and 35:1 for the 100,000 ppm initial salinity. Right plots: 2D cross-sections for the gas saturation, which is the mass fraction of exsolved CO_2_ in the pore fluids, for the three salinities after 50 and 100 years of carbonated water injection.

Figures S7 and S8 illustrate 2D cross-section time snapshots for the CO_2_ mass fraction in the liquid, gas saturation, water density, and salinity for the 10,000 and 100,000 ppm initial salinity systems when injecting CO_2_ charged water with a seawater salinity of 35,000 ppm. Figure 5 compares the model results for the three initial salinities after 50 and 100 years of carbonated water injection. Lowering the salinity results in an improved storage capacity as indicated by the CO_2_ concentration profiles in Fig. 5. However, the injected water to CO_2_ ratio was the same for the 10,000- and the 35,000 ppm initial salinity calculations. Decreasing the ratio to 24:1 results in CO_2_ gas escaping to the surface even for 10,000 ppm initial salinity. For a 100,000 ppm salinity, it was not possible to inject 100,000 metric tons/year of CO_2_ for 100 years without CO_2_ gas escaping to the surface, as demonstrated in the 2D cross-sections for the gas saturation after 100-years in Fig. 5. The density of 100,000 ppm saline water is approximately 1050 kg/m^3^ near the injection zone, which is denser than that of the injected carbonated seawater with a density of approximately 1020 kg/m^3^. This means that solubility trapping by injecting saline carbonated water having a significantly lower salinity than the formation water is not an effective trapping mechanism, as the injected carbonated water prefers to flow upwards towards the surface, as shown in Fig. 5. Reducing the CO_2_ concentration in the injected water does not help, as lowering the CO_2_ concentration of the injected water further reduces the density of the injected water and increases the convection upwards towards the surface. A demonstration of carbonated water movement upwards during and after injection stops after 50 years of injection into 100,000 ppm saline reservoir can be seen in Fig. S9.

When the injected water has the same salinity as the formation water, the storage potential for the 10,000 ppm system improves, and solubility storage in the 100,000 ppm system is possible by the injection of CO_2_ charged water. The minimum water to CO_2_ ratio waters having salinities of 10,000, and 100,000 ppm, respectively, without CO_2_ escaping to the surface are 22:1 and 35:1, respectively. This reflects the decrease in CO_2_ solubility due to increasing salinity^57^. The overall flow behavior for both systems is similar to the reference system. In each of these cases, the carbonated water is denser and tends to flow downwards.

### Impact of salinity gradient on carbon storage and security

The effect of the presence of a salinity gradient in the reservoir on the fate of injected water-dissolved CO_2_ injection is considered assuming the presence of an initial 10 and 100 ppm/m salinity gradient. The results of these model calculations are shown in Figs. S10,S11. A salinity gradient of 10 ppm/m results in a lower salinity throughout the reservoir compared to the reference system. The storage capacity of this system is identical to that of the reference system as the injection water has a salinity of 35,000 ppm, the same as the reference system. A salinity gradient of 100 ppm/m results in significantly higher salinity with depth compared to the reference system, reaching 200,000 ppm at a depth of 2000 m. As in the case of the lower salinity gradient system, the storage capacity in this system is dictated by the salinity of the injected water (35,000 ppm). The high salinity gradient introduces a fluid density gradient that increases with increased depth, which helps push the injected carbonated water downwards towards the bottom of the system, as shown in Figs. S11,S12.

**Figure 6:**
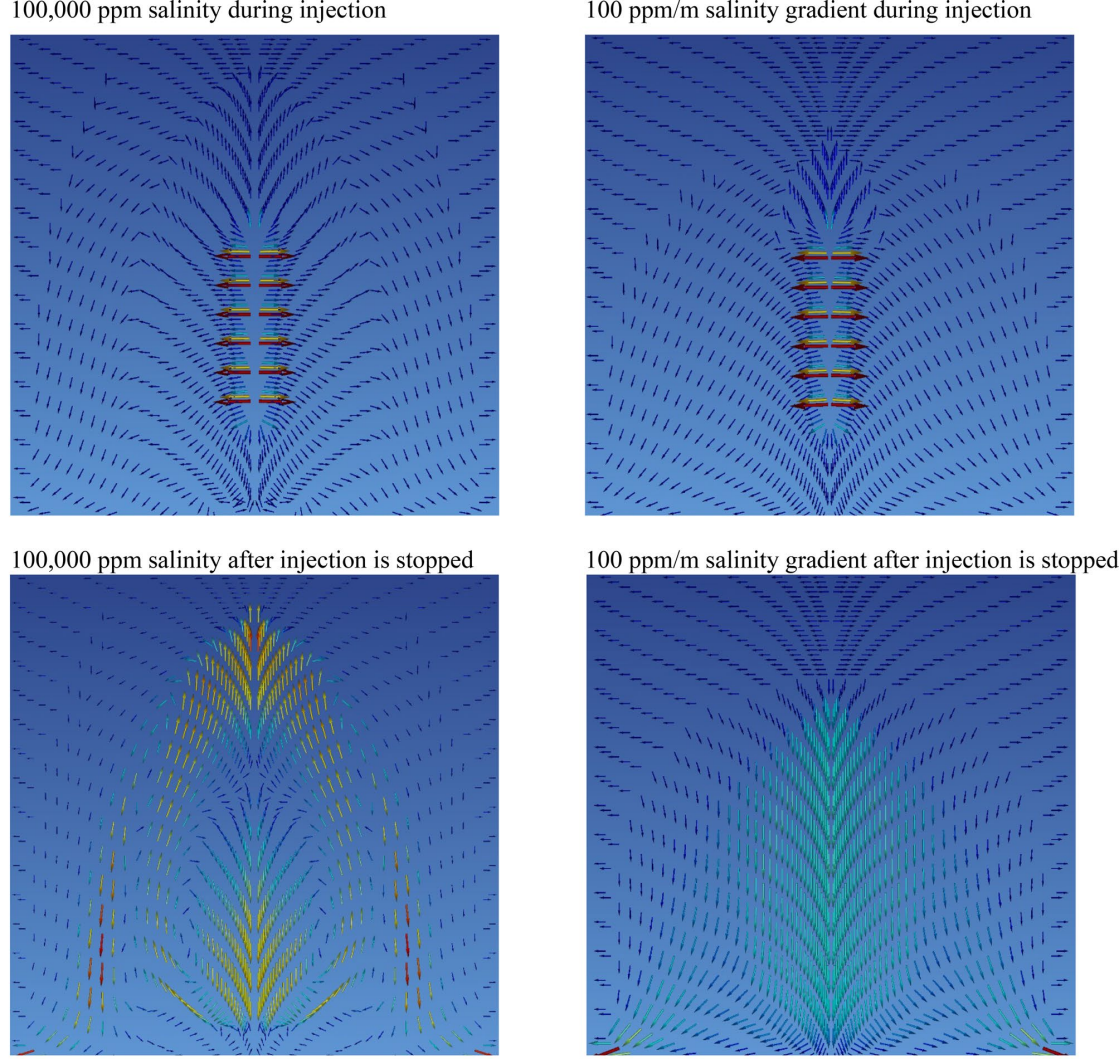
Comparison of the dissolved CO_2_ flow paths during the final stages of injection and after the injection has stopped. The left plots represent the flow paths in a system having a constant initial salinity of 100,000 ppm. The right plots represent the flow paths in a system with an initial salinity gradient of 100 ppm/m. Carbonated water was injected for 50 years for the constant initial salinity of 100,000 ppm, and for 100 years for the initial salinity gradient of 100 ppm/m. The non-blue colors highlight the higher flow rate zones, and the arrows indicate the flow direction.

Figure 6 illustrates the fluid flow paths during and after carbonated water injection into a system with initial constant 100,000 ppm salinity and for a system having a 100 ppm/m salinity gradient. Both during fluid injection and after the injection, the majority of CO_2_ near the injection zone travels upwards in the constant 100,000-ppm initial salinity system. In contrast, the majority of CO_2_ travels downwards during injection for the 100 ppm/m salinity gradient, and the CO_2_ solely travels downwards after the end of its injection.

### Impact injection zone thickness and injection depth on carbon storage and security

The impact of injection zone thickness was explored by considering thicknesses of 400- and 1200 m and comparing model results to the reference system of 800 m. The results of these model calculations are shown in Figs. S13,S14. Increasing the thickness of the injection zone to 1200 m does not improve the storage capacity compared to the reference system. Decreasing the thickness of the injection zone to 400 m slightly reduces the storage capacity, as it requires a 26:1 water to CO_2_ ratio, compared to 25:1 for the reference system to avoid the escape of CO_2_ to the surface. This is due to the increased pressure in the injection zone in the 400 m case, which drives more of the carbonated water upwards towards the surface as shown in Fig. S13. Note that the risk of fracturing due to pressure buildup may increase with decreased injection zone thickness.

Changing the injection depth has a clearer impact on storage potential compared to the thickness of the injection zone. A deeper injection results in a better storage capacity due to the increased CO_2_ solubility with increasing depth and the presence of an open flow boundary at the margins of the modelled system. For example, injecting at a depth of 1000 to 1800 m (with an 800 m thick injection zone) improves the minimum water to CO_2_ ratio to 22:1, and injecting in at a depth of 1700 to 2000 m (a 300 m thick injection zone) improves the minimum water to CO_2_ ratio to 19:1. The results of these model calculations are shown in Figs. S15,S16. In this case, the CO_2_ storage capacity is improved due to injection depth despite the smaller perforation thickness.

### Impact of horizontal to vertical permeability ratio on carbon storage and security

**Figure 7:**
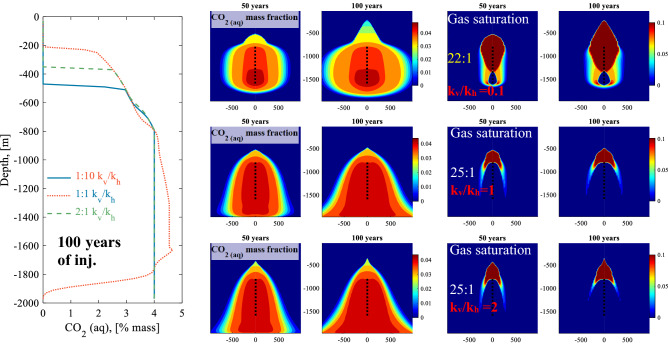
Left plot: Comparison of CO_2_ concentration profiles at the well versus depth after 100 years of injection for systems having k_v_/k_h_ ratios of 1:10, 1:1 and 2:1. The water to CO_2_ injection mass ratio is 22:1 for the k_v_/k_h_ ratio of 1:10, and 25:1 for the k_v_/k_h_ ratio of 1:1 (reference system), and k_v_/k_h_ = 2:1 as indicated in the plots.Center and right plots: 2D cross-sections for the CO_2_ mass fraction and gas saturation, which is the mass fraction of exsolved CO_2_ in the pore fluids, for the three modelled systems after 50 and 100 years of carbonated water injection.

The effect of heterogeneous permeability on the fate of water dissolved CO_2_ injection was assessed by altering the ratio between the vertical and horizontal permeability (k_v_/k_h_) from the homogeneous reference system. Three cases were considered: systems having a k_v_/k_h_ of 0.1, 1 (reference system) and 2. To this end, the horizontal permeability was held constant, and the vertical permeability was changed to 10 and 200 mD in the modelled systems. Figure 7 shows a comparison of the fate of CO_2_ injected into the systems having the three permeability ratios. The k_v_/k_h_ = 0.1 system restricts fluid flow from moving in the vertical direction, creating more preferential flow paths in the horizontal direction, as seen by the 2D cross-section for aqueous CO_2_% mass in Fig. 7. The preferred horizontal movement results in a better storage security allowing for a reduced water to CO_2_ injection mass ratio of 22:1. In addition, the enhanced horizontal movement might improve the effective storage capacity as larger portion of the midsection of the reservoir is accessed compared with the reference system. In contrast, the k_v_/k_h_ = 2 system induces preferential flow in the vertical direction, resulting in enhanced CO_2_ exsolution, as seen in Fig. 7. The k_v_/k_h_ = 2 system requires the same water to CO_2_ ratio as the reference system with k_v_/k_h_ = 1 to avoid CO_2_ leakage to the surface. In this case, the denser CO_2_-charged water enhances the flow path downwards, compensating for the limited flow in the horizontal direction.

## Discussion

The results described above suggest that the injection of water dissolved CO_2_ can provide significant and safe long-term subsurface CO_2_ storage even in the absence of traditional caprocks. Such results potentially open the possibility of using unconfined subsurface systems for the solubility storage of CO_2_ injected as a water dissolved phase around the world. Notably, the impact of most physical parameters changes little this result.

The impact of the various physical parameters considered in this study are Table 1. Most of the investigated scenarios showed similar maximum injection water to CO_2_ mass ratios ranging from 20 to 30. The calculated results suggest that the temperature and salinity of the injected CO_2_ charged water dictated the storage potential more than these parameters in the original reservoir fluids. This behavior is because fluid injection cools and dilutes the reservoir fluids over time. The storage security of the system, however, is highly dependent on the initial reservoir temperature and salinity gradients. These factors impact the fluid flow direction of the injected fluid. In the most extreme situation, the injection of a mildly saline CO_2_-charged water into a highly saline reservoir could introduce upwards convective flow. The depth of the injection zone also plays a significant role on the solubility storage potential, assuming hydrostatic pressure^58^. Injecting in a deeper interval improves the CO_2_ solubility, thus reducing the required amount of injection water required. Injecting deeper, however, might introduce higher wellbore pressure because of potentially lower formation permeability and fluid friction effect within the well, which was not accounted for in this study. Furthermore, the injectivity cost might increase with increased injection depth^59^. The impact of reservoir heterogeneity in terms of permeability anisotropy showed improved storage potential with higher horizontal to vertical ratio.

The calculations in this study illustrate the potential for subsurface solubility carbon storage in unreactive unconfined aquifers by the injection of carbonated water. Note that the current study considered only a simplified systems that had no provision for capillary or mineral trapping. These trapping mechanisms, when accounted for, will provide additional storage potential. This choice was made in this study to focus directly on solubility trapping as a primary storage mechanism to define the temperature, pressure, salinity, and permeability limits on the fate of water dissolved CO_2_ in the subsurface. Other factors could also come to play in real subsurface systems. For instance, porosity often decreases with depth in sandstone reservoirs^53,60^. The rock permeability tends to decrease as porosity decreases^61^. The effect of subsurface heterogeneities such as layering, salt bodies, and dipping strata can have a profound effect on the subsurface flow dynamics and storage efficiency^62^. Note that all calculations performed in this study assumed the original formation waters are CO_2_ free. Most natural subsurface aquifers, however, contain at least some dissolved CO_2_^63^. Other factors not considered directly in the calculations presented in this study include the effects of regional flow patterns, wellbore pressure, and subsurface mineral-fluid interaction. Each factor may have an effect on subsurface carbon storage mechanisms. The impact of these processes should be considered and examined in detail for each individual subsurface system scenario.

The capital and operational costs and dissolution efficiency are key factors when assessing different engineering solutions, including the injection of carbonated water into the subsurface^13^. The dissolution of CO_2_ into water at the surface, followed by its injection into the subsurface has been considered by Burton and Bryant^11^. Their analysis concluded that compared to supercritical CO_2_ injection, the capital costs increase by 60%, and additional energy consumption is anticipated during operation for surface CO_2_ dissolution and the subsequent injection of this fluid. This process was modified and implemented as part of the CarbFix2 project, where above surface water dissolution was used to capture CO_2_ from an impure exhaust stream^64^. In this case, the use of surface dissolution yielded substantial cost savings compared to alternative CO_2_ capture approaches. Alternatively, wellbore dissolution of CO_2_ into water has been proposed and implemented as part of the original CarbFix1 project^14,65^. This approach combines the engineering benefits of surface dissolution while avoiding most extra costs for cases where a pure CO_2_ stream is available^13^. Wellbore dissolution, compared to supercritical CO_2_ injection, has a lower operating cost per well with up to one-third less energy consumed during compression^66^. In some cases, CO_2_ can just be added to existing waste water streams. This could lead to subsurface solubility storage of CO_2_ with limited additional cost. There are more than 180,000 wells for oil and gas applications in the United States and more than 500,000 wells for other applications^67^. More than 9 million m^3^/day of waste water from oil and gas operation are injected in the United States. This corresponds to more than 3 Gigatons of fluid per year^68^. Adding CO_2_ to the existing waste water streams in the United States alone has the potential to store tens to hundreds of megatons of CO_2_ annually at little extra cost.

Alternatively, dedicated wells could be drilled for the injection of CO_2_ charged water for subsurface solubility storage. The injection of CO_2_ charged water requires injection of a substantially larger mass than the injection of a pure CO_2_ stream. This will likely limit the overall mass of CO_2_ that can be injected into each well, so therefore more wells would be required for this carbon storage approach^17^. Bodnar et al.^69^, however, concluded that the formation volume needed to store dissolved CO_2_ is less than the formation volume needed to store equivalent amount as supercritical CO_2._ Moreover, Burton and Bryant^70^, suggested that less net formation volume is required to extract and re-inject formation water with dissolved CO_2_ compared to net formation volume needed for separate scCO_2_ phase. Burton and Bryant^11^ demonstrated a theoretical case study on how to store CO_2_ emissions from a 500-MW power plant near Mt. Simon Formation in the US. They suggested the use of 50 injection and 50 extraction wells to store 3.65 Mt CO_2_ annually, equivalent to 90% of the power plants emissions. At least some of the costs of such required additional wells may be overcome because (1) the depth of CO_2_ injection can be less for the injection when this gas is in the aqueous phase as opposed to a supercritical phase, and (2) the injection into hydrostatically pressured systems require less compression or wells that can support high pressure^71^.

The model calculations in the present study are based on the injection of 100,000 metric tons of CO_2_ into the subsurface annually. The maximum injection rate will be site dependent, which is a function of depth, wellbore size, reservoir connectivity, permeability, and porosity of the subsurface formation.. Nevertheless, an annual injection of 100,000 metric tons CO_2_ is approximately equivalent to the total emissions of a reverse-osmosis desalination plant, producing desalinated water at a rate of 30 million cubic meters per year^72,73^. Furthermore, according to the 2021 Global Status of CCS Report^74^, a significant number of operating a planned CCS facilities that have an annual injection capacity of around 100,000 metric tons CO_2_. For example, an annual operational CO_2_ storage capacity of 59,000 metric tons is reported for a CO_2_ EOR facility in Hungary, and an annual operational CO_2_ storage capacity of 100,000 metric tons, for Karamay Dunhua Oil Technology CCUS EOR project in China. The list also includes five planned Biorefinery Carbon Capture and Storage project in the US, all with a projected annual storage capacity around 100,000 metric tons. Multiple wells would be required to store larger quantities of CO_2_ from larger emission sources. For example, the Gorgon Carbon Dioxide Injection project in Australia includes 17 wells^75^. However, large emission sources often have numerous existing water disposals wells nearby that can potentially be incorporated in the carbon storage strategy to reduce the initial cost^68^.

## Conclusions

The presented results encourage further consideration of solubility trapping in unconfined subsurface aquifers, potentially unlocking significant CO_2_ storage capacity. This would make CO_2_ storage more accessible at different locations around the world in systems without caprocks. In this study, the only scenario in which the injected CO_2_ was not securely stored was the case in which the injected carbonated sea water was of much lower density than the in-situ water. All other scenarios investigated suggest that there is substantial potential for the safe long-term storage of CO_2_ by solubility trapping.

The injection of CO_2_-charged water has the advantage of requiring less compression and shallower, cheaper wells, thus resulting in a comparable storage cost to the injection of supercritical CO_2_. The need for large volumes of water can be overcome by using seawater or withdrawing and reinjecting in-situ water from the same formation. Furthermore, the withdrawal wells can be used to monitor the fate of the injected CO_2_.

The process of water-dissolved CO_2_ injection into hydrostatically pressured subsurface systems eliminates the need for a structural or stratigraphic seal, assuming fixed pressure open boundaries. Unconfined reservoirs are more abundant than confined reservoirs and can potentially be found in closer proximity to major carbon emitting sources. This significantly increases the number of potential subsurface carbon storage sites around the world. Furthermore, solubility trapping promotes carbon mineralization resulting in higher storage potential. Although the structures and hydraulics of many hydrostatically pressured saline aquifers have not yet been studied in detail, the fact that water-charged CO_2_ tends to sink overcomes the need for extended knowledge on the subsurface geology prior to injection into a potential storage site. Overall, the results of this work further compel the consideration of carbonated water injection into unconfined aquifers, a generally overlooked potential carbon storage host, for the long-term storage of carbon-dioxide.

## Supplementary Information

Supplementary Figures.

## Data Availability

The datasets used and/or analyzed during the current study available from the corresponding author on reasonable request.
