# Supplementary material for: Assessing the potential of solubility trapping in unconfined aquifers for subsurface carbon storage
Source: Sci Rep. 2022 Nov 28;12:20452. doi: 10.1038/s41598-022-24623-6 (PMC9705318; doi:10.1038/s41598-022-24623-6)

# Assessing the potential of solubility trapping in unconfined aquifers for subsurface carbon storage

Mouadh Addassi[a*], Abdirizak Omar[a], Hussein Hoteit[a], Abdulkader M. Afifi[a], Serguey Arkadakskiy[b], Zeyad T. Ahmed[b], Noushad Kunnummal[b], Sigurdur R. Gislason[c], Eric H. Oelkers[d]

[a] King Abdullah University of Science and Technology (KAUST), Thuwal 23955-6900, Saudi Arabia.
[b] Environmental Protection, Saudi Arabian Oil Company, Dhahran, Saudi Arabia
[c] Institute of Earth Sciences, University of Iceland, Sturlugötur 7, 102 Reykjavík, Iceland
[d] Géosciences Environnement Toulouse (GET), CNRS UMR 5563, France.

* Corresponding author email address: mouadh.addassi@kaust.edu.sa

## SUPPLEMENTARY INFORMATION

S1. Averaged annual re-injection rates per well for various geothermal projects. The rate per well was calculated from the total injection rate and the number of wells. Data collected by Hoteit et al. [42].

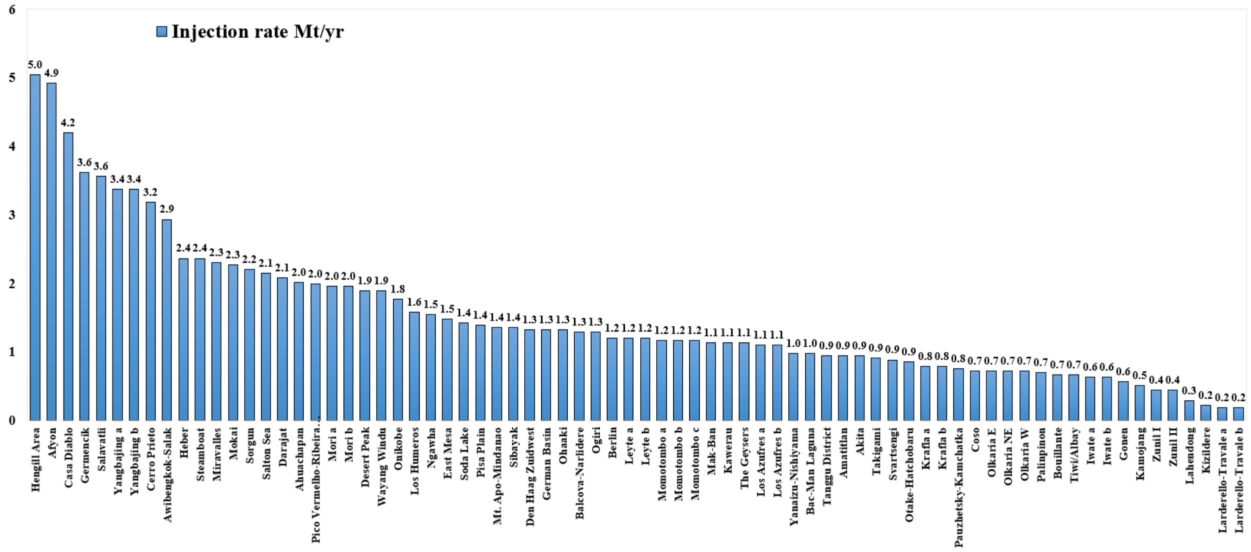

S2. Calculated $CO_2$ concentrations in the subsurface liquid and exsolved $CO_2$ mass fraction in the pore fluids 10, 100 years of carbonated water injection and 10 and 100 years after the end of the injection. The results shown are for the 'reference system'. This system has a 30 °C/km gradient, a constant salinity of 35,000 ppm, equal to that of seawater, and water injection temperature of 40 °C. The water to $CO_2$ injection mass ratio is 25:1. The upper plots show the 2D cross sections of the $CO_2$ concentration in the liquid phase whereas the lower plots show 2D cross sections of the $CO_2$ gas saturation, which is the mass fraction of exsolved $CO_2$ in the pore fluids at the indicated times. Color bars defining the range for each of the properties are displayed on right side. The dotted line marks the injection zone.

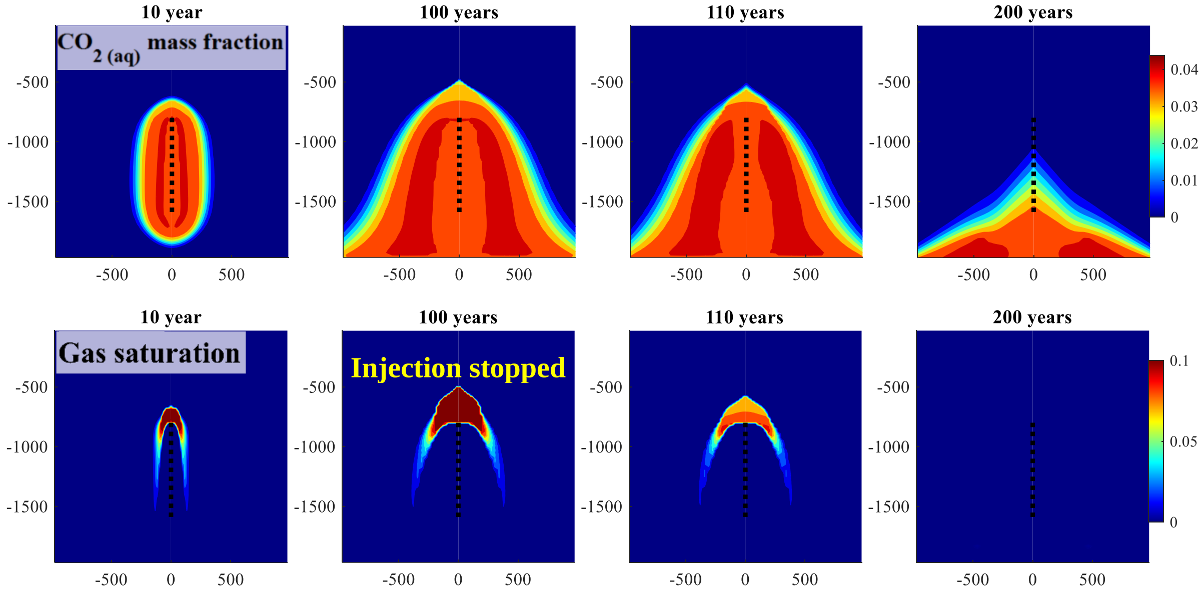

S3. Calculated $CO_2$ concentrations in the subsurface liquid and exsolved $CO_2$ mass fraction in the pore fluids after 1, 10, 30 and 40 years of carbonated water injection. The results shown are for the 'reference system'. This system has a 30 °C/km gradient, a constant salinity of 35,000 ppm, equal to that of seawater, and water injection temperature of 40 °C. The water to $CO_2$ injection mass ratio is 24:1. The upper plots show the 2D cross sections of the $CO_2$ concentration in the liquid phase whereas the lower plots show 2D cross sections of the $CO_2$ gas saturation, which is the mass fraction of exsolved $CO_2$ in the pore fluids, at the indicated times. Color bars defining the range for each of the properties are displayed on right side. The dotted line marks the injection zone. Exsolved $CO_2$ reaches the surface after approximately 40 years of carbonate water injection.

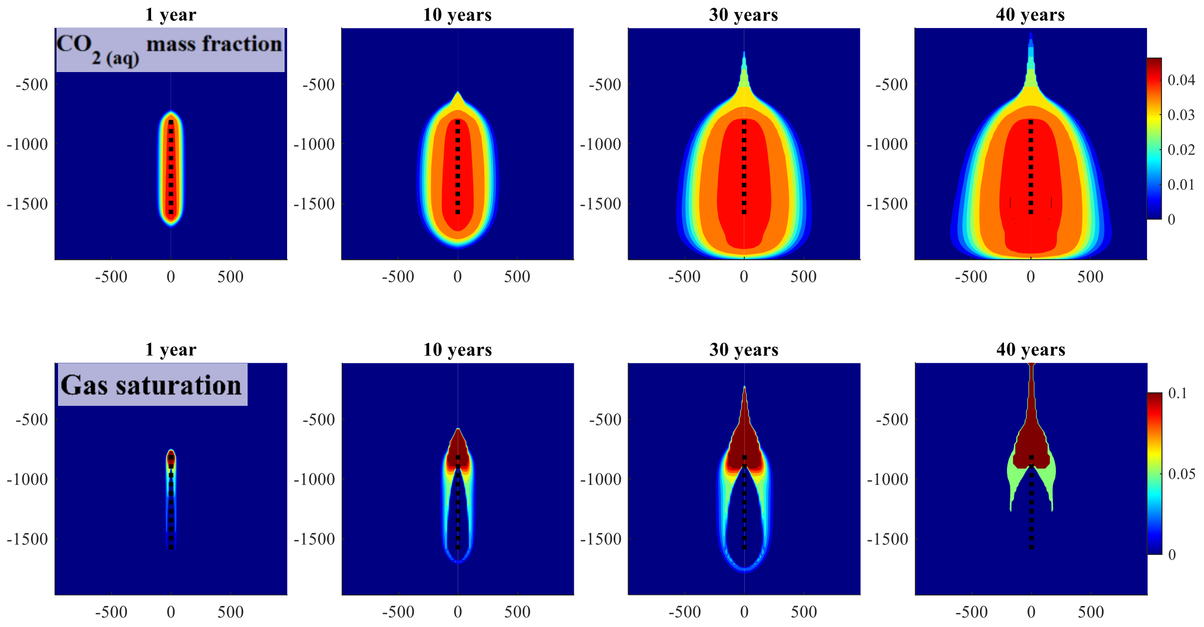

S4. Calculated $CO_2$ concentrations in the subsurface liquid exsolved $CO_2$ mass fraction in the pore fluids after 1, 10, 50 and 100 years of carbonated water injection. The results shown are for the 'isothermal system'. This system has a 30 ˚C/km gradient, a constant salinity of 35000 ppm, equal to that of seawater. We exclude the impact of water injection temperature in this example, assuming isothermal reservoir conditions. The water to $CO_2$ injection mass ratio needed to prevent exsolved $CO_2$ from reaching the surface is 28:1. The upper plots show the 2D cross sections of temperature, followed by the $CO_2$ concentration in the liquid phase, $CO_2$ gas saturation, which is the mass fraction of exsolved $CO_2$ in the pore fluids, whereas the lower plots show 2D cross sections of the density of the liquid phase at the indicated times. Color bars defining the range for each of the properties are displayed on right side. The dotted line marks the injection zone.

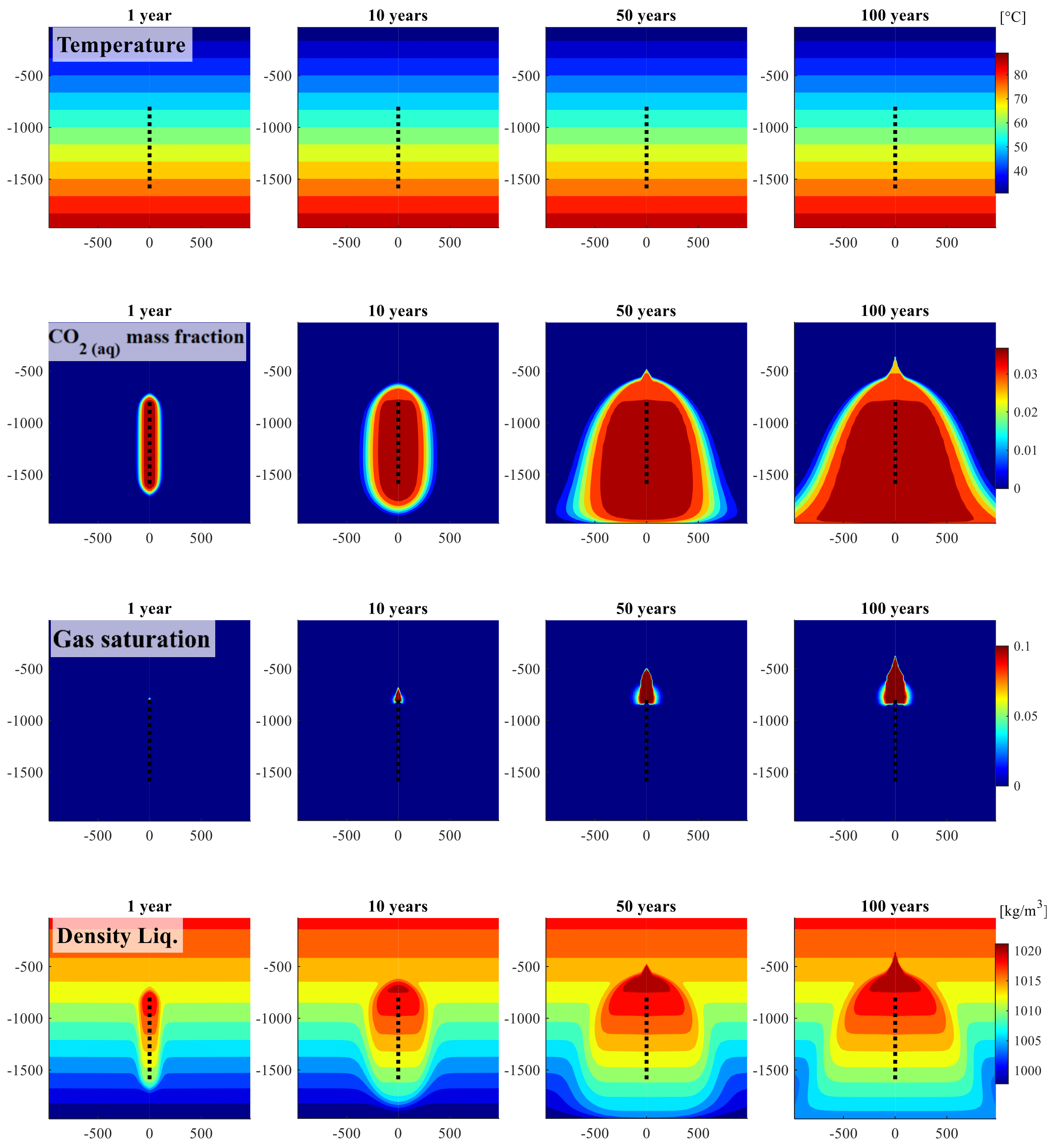

S5. Calculated $CO_2$ concentrations in the subsurface liquid exsolved $CO_2$ mass fraction in the pore fluids after 1, 10, 100 and 100 years of carbonated water injection. The results shown are for the ‘18 °C/km temperature gradient system’. This system has a 18 °C/km gradient, a constant salinity of 35000 ppm, equal to that of seawater, and water injection temperature of 40 °C. The water to $CO_2$ injection mass ratio is 25:1. The upper plots show the 2D cross sections of temperature, followed by the $CO_2$ concentration in the liquid phase, $CO_2$ gas saturation, which is the mass fraction of exsolved $CO_2$ in the pore fluids, whereas the lower plots show 2D cross sections of the density of the liquid phase at the indicated times. Color bars defining the range for each of the properties are displayed on right side. The dotted line marks the injection zone.

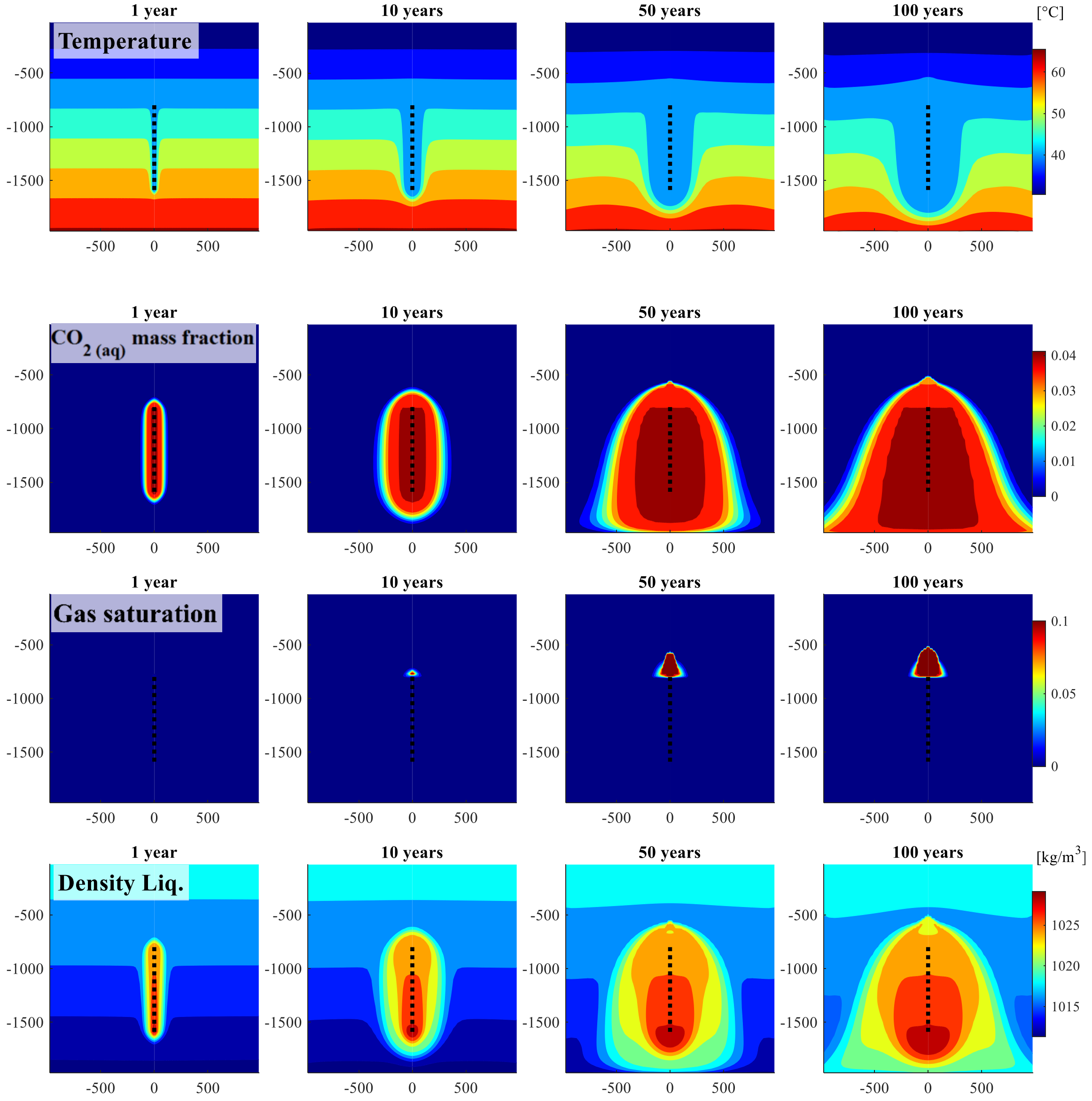

S6. Calculated $CO_2$ concentrations in the subsurface liquid exsolved $CO_2$ mass fraction in the pore fluids after 1, 10, 100 and 100 years of carbonated water injection. The results shown are for the '50 °C/km temperature gradient system'. This system has a 50 °C/km gradient, a constant salinity of 35,000 ppm, equal to that of seawater, and water injection temperature of 40 °C. The water to $CO_2$ injection mass ratio is 26:1. The upper plots show the 2D cross sections of temperature, followed by the $CO_2$ concentration in the liquid phase, $CO_2$ gas saturation, which is the mass fraction of exsolved $CO_2$ in the pore fluids, whereas the lower plots show 2D cross sections of the density of the liquid phase at the indicated times. Color bars defining the range for each of the properties are displayed on right side. The dotted line marks the injection zone.

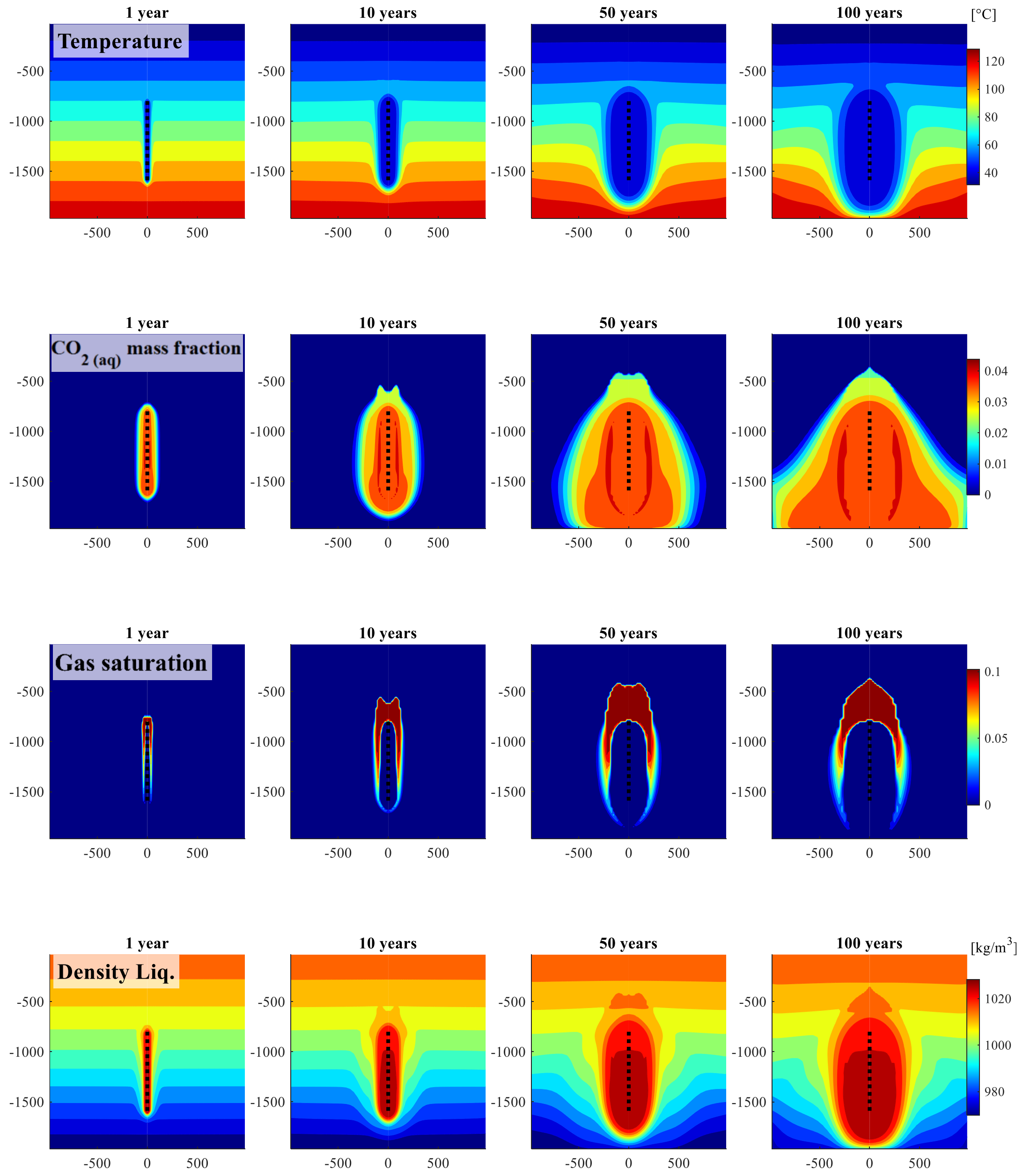

S7. Calculated $CO_2$ concentrations in the subsurface liquid exsolved $CO_2$ mass fraction in the pore fluids after 1, 10, 100 and 100 years of carbonated water injection. The results shown are for the '10,000 ppm initial salinity system'. This system has a 30 °C/km gradient, a constant initial reservoir salinity of 10,000 ppm, injection water salinity of 35,000 ppm and temperature of 40 °C. The water to $CO_2$ injection mass ratio is 25:1. The upper plots show the 2D cross sections of salinity (NaCl mass fraction in the liquid), followed by the $CO_2$ concentration in the liquid phase, $CO_2$ gas saturation, which is the mass fraction of exsolved $CO_2$ in the pore fluids, whereas the lower plots show 2D cross sections of the density of the liquid phase at the indicated times. Color bars defining the range for each of the properties are displayed on right side. The dotted line marks the injection zone.

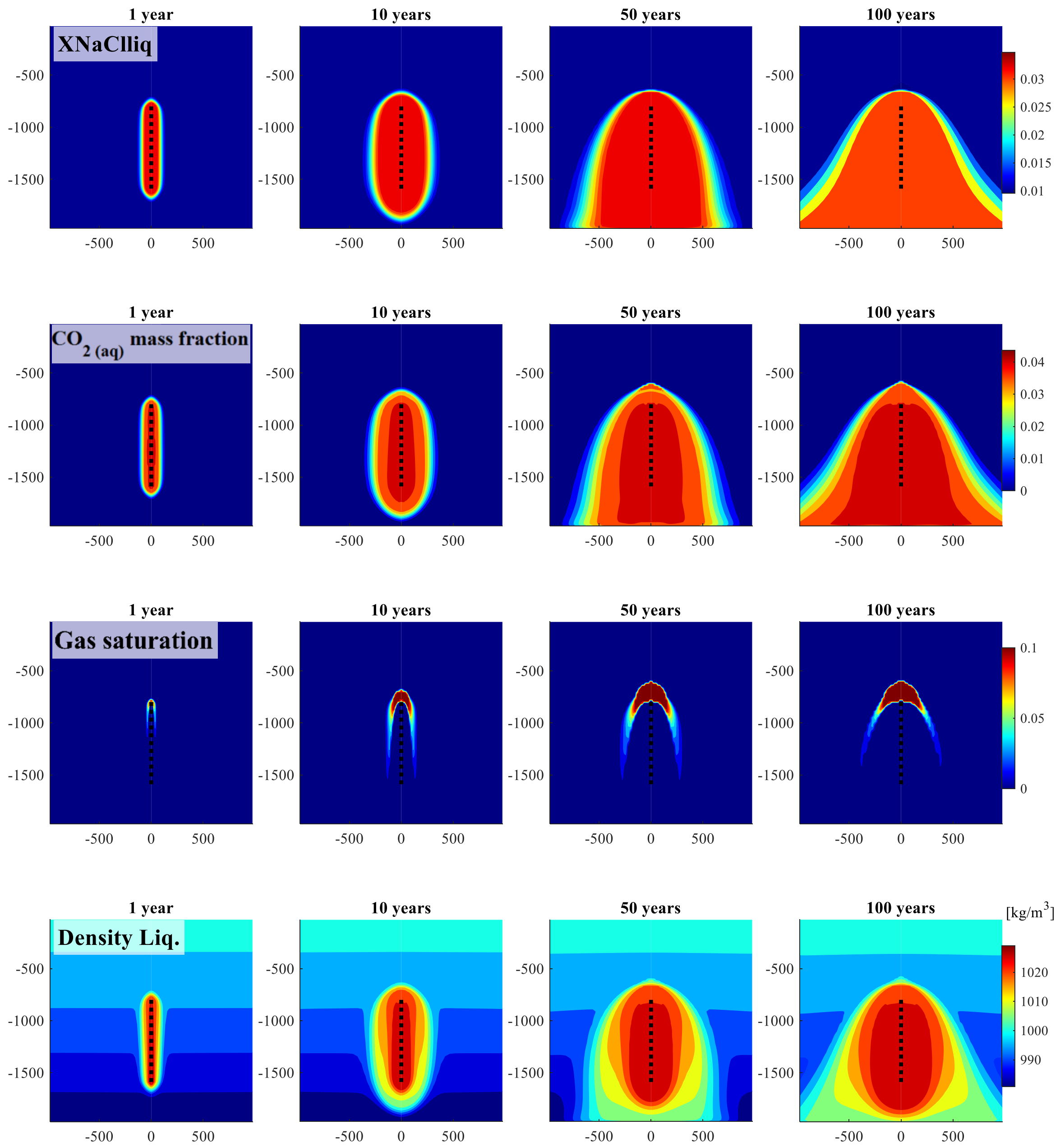

S8. Calculated $CO_2$ concentrations in the subsurface liquid exsolved $CO_2$ mass fraction in the pore fluids after 1, 10, 100 and 100 years of carbonated water injection. The results shown are for the '100,000 ppm initial salinity system'. This system has a 30 °C/km gradient, a constant initial reservoir salinity of 100,000 ppm, injection water salinity of 35,000 ppm and temperature of 40 °C. The water to $CO_2$ injection mass ratio is 38:1. The upper plots show the 2D cross sections of salinity (NaCl mass fraction in the liquid), followed by the $CO_2$ concentration in the liquid phase, $CO_2$ gas saturation, which is the mass fraction of exsolved $CO_2$ in the pore fluids, whereas the lower plots show 2D cross sections of the density of the liquid phase at the indicated times. Color bars defining the range for each of the properties are displayed on right side. The dotted line marks the injection zone. Results show that this injection would likely lead to $CO_2$ leakage.

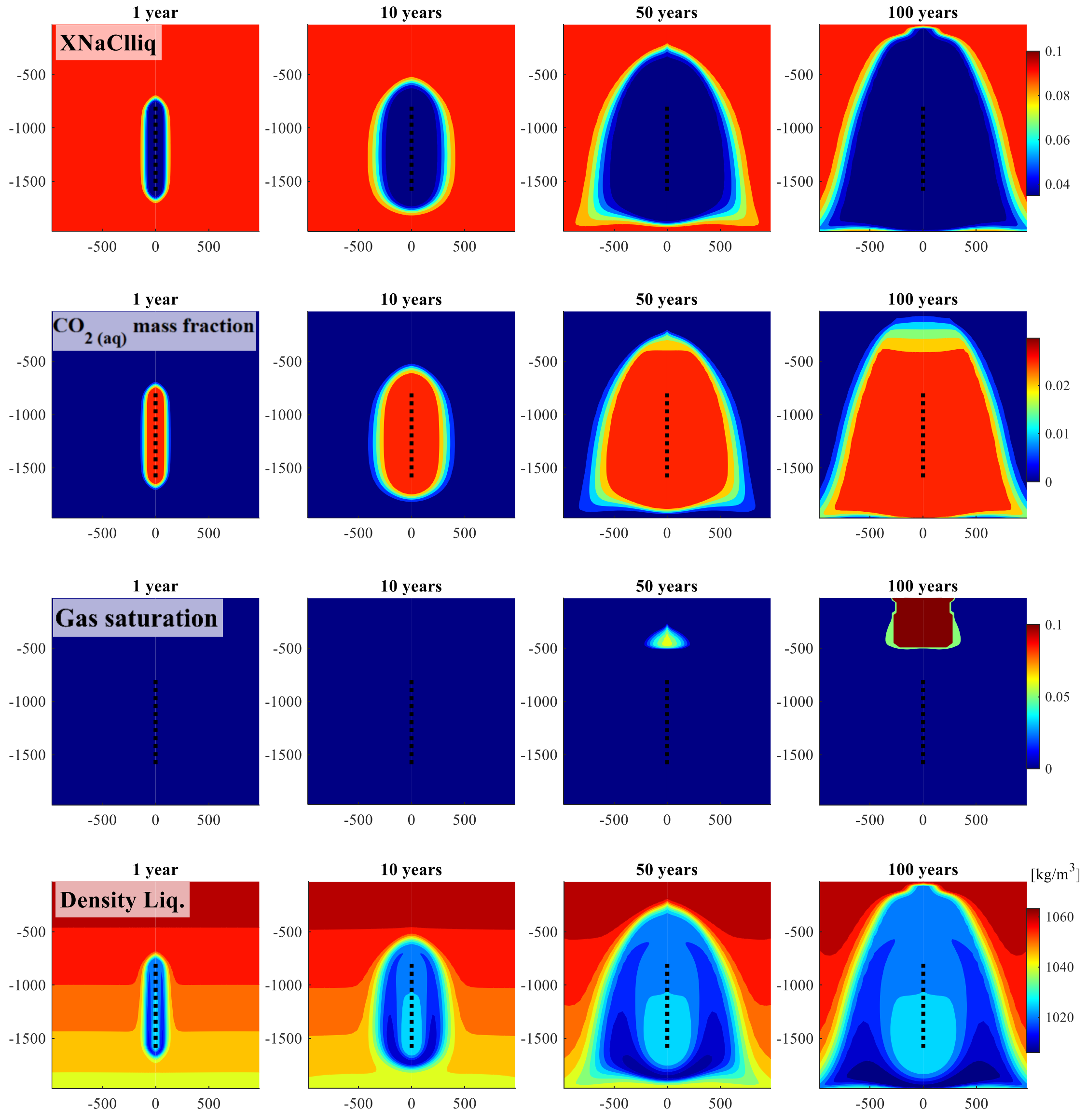

S9. Calculated $CO_2$ concentrations in the subsurface liquid exsolved $CO_2$ mass fraction in the pore fluids after 10, 50 years of carbonated water injection and 50 and 200 years after the end of the injection. The results shown are for the '100,000 ppm initial salinity system'. This system has a 30 ˚C/km gradient, a constant initial reservoir salinity of 100,000 ppm, injection water salinity of 35,000 ppm and temperature of 40 ˚C. The water to $CO_2$ injection mass ratio is 38:1. The upper plots show the 2D cross sections of $CO_2$ concentration in the liquid phase, followed by $CO_2$ gas saturation, which is the mass fraction of exsolved $CO_2$ in the pore fluids, whereas the lower plots show 2D cross sections of the density of the liquid phase at the indicated times. Color bars defining the range for each of the properties are displayed on right side. The dotted line marks the injection zone. Results show that this injection would likely lead to $CO_2$ leakage.

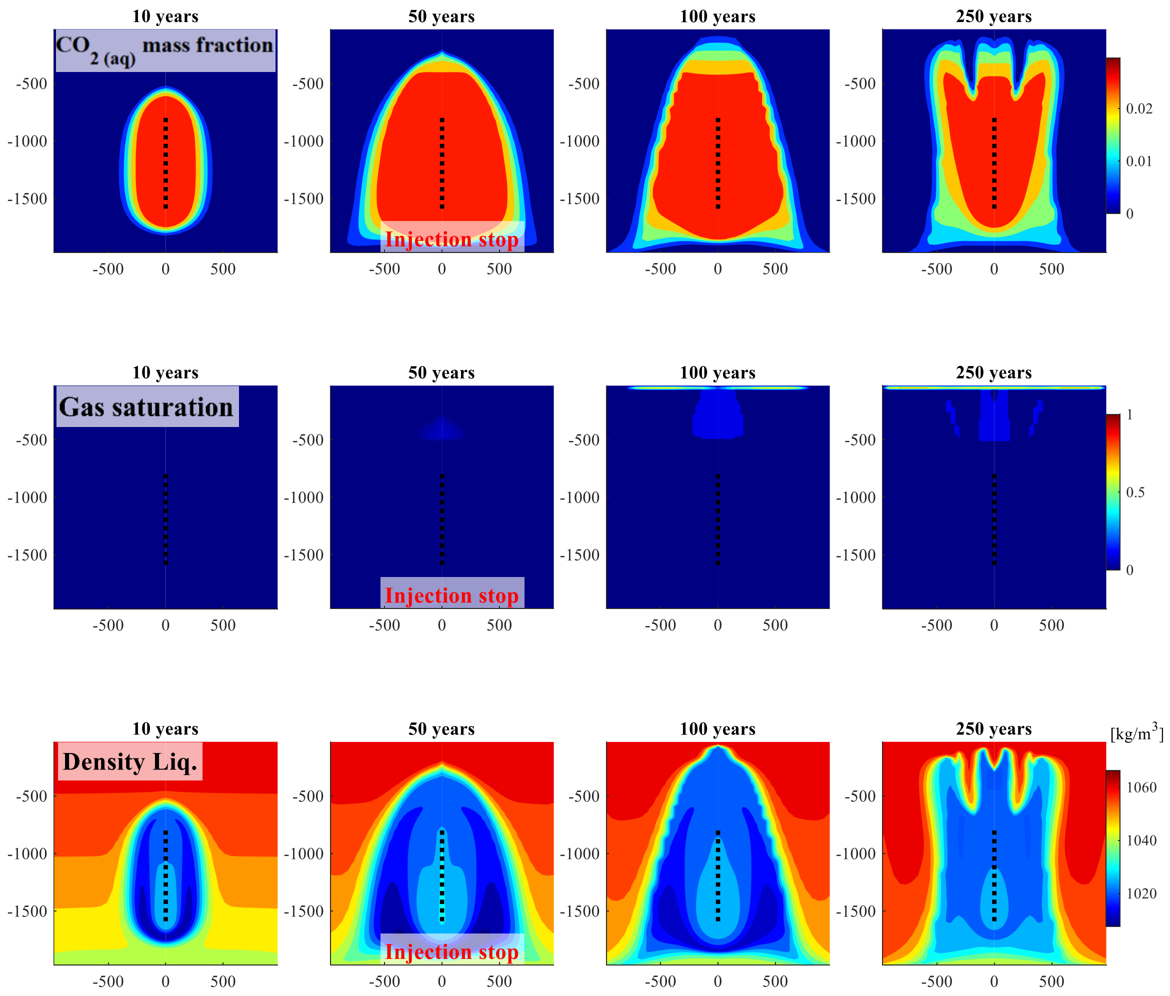

S10. Calculated $CO_2$ concentrations in the subsurface liquid exsolved CO2 mass fraction in the pore fluids after 1, 10, 100 and 100 years of carbonated water injection. The results shown are for the '10 ppm/m initial salinity gradient system'. This system has a 30 °C/km gradient, a 10 ppm/m initial reservoir salinity, injection water salinity of 35,000 ppm and temperature of 40 °C. The water to $CO_2$ injection mass ratio is 25:1. The upper plots show the 2D cross sections of salinity (NaCl mass fraction in the liquid), followed by the $CO_2$ concentration in the liquid phase, $CO_2$ gas saturation, which is the mass fraction of exsolved $CO_2$ in the pore fluids, whereas the lower plots show 2D cross sections of the density of the liquid phase at the indicated times. Color bars defining the range for each of the properties are displayed on right side. The dotted line marks the injection zone.

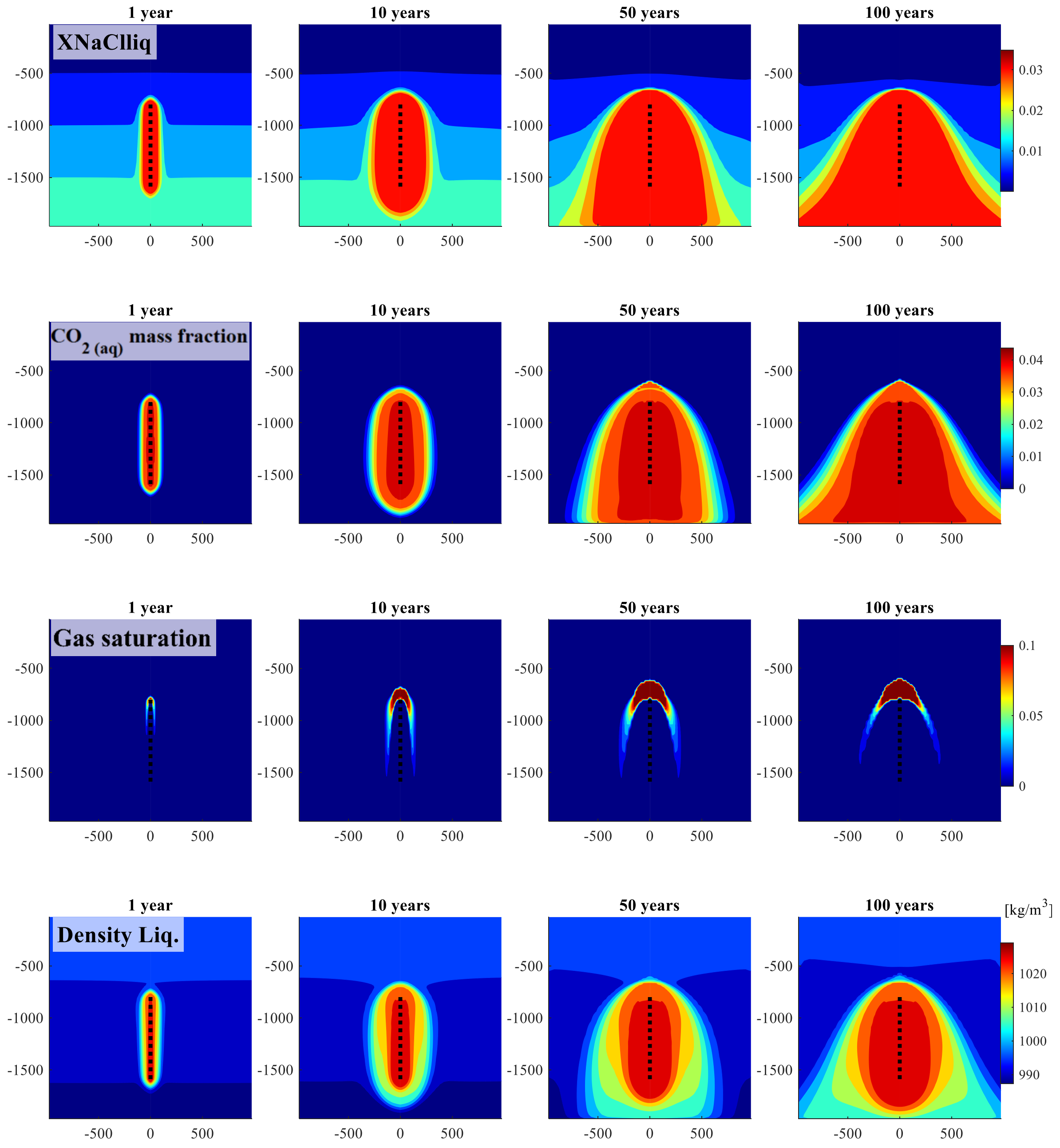

S11. Calculated $CO_2$ concentrations in the subsurface liquid exsolved $CO_2$ mass fraction in the pore fluids after 1, 10, 100 and 100 years of carbonated water injection. The results shown are for the ‘100 ppm/m initial salinity gradient system’. This system has a 30 °C/km gradient, a 100 ppm/m initial reservoir salinity, injection water salinity of 35,000 ppm and temperature of 40 °C. The water to $CO_2$ injection mass ratio is 25:1. The upper plots show the 2D cross sections of salinity (NaCl mass fraction in the liquid), followed by the $CO_2$ concentration in the liquid phase, $CO_2$ gas saturation, which is the mass fraction of exsolved $CO_2$ in the pore fluids, whereas the lower plots show 2D cross sections of the density of the liquid phase at the indicated times. Color bars defining the range for each of the properties are displayed on right side. The dotted line marks the injection zone.

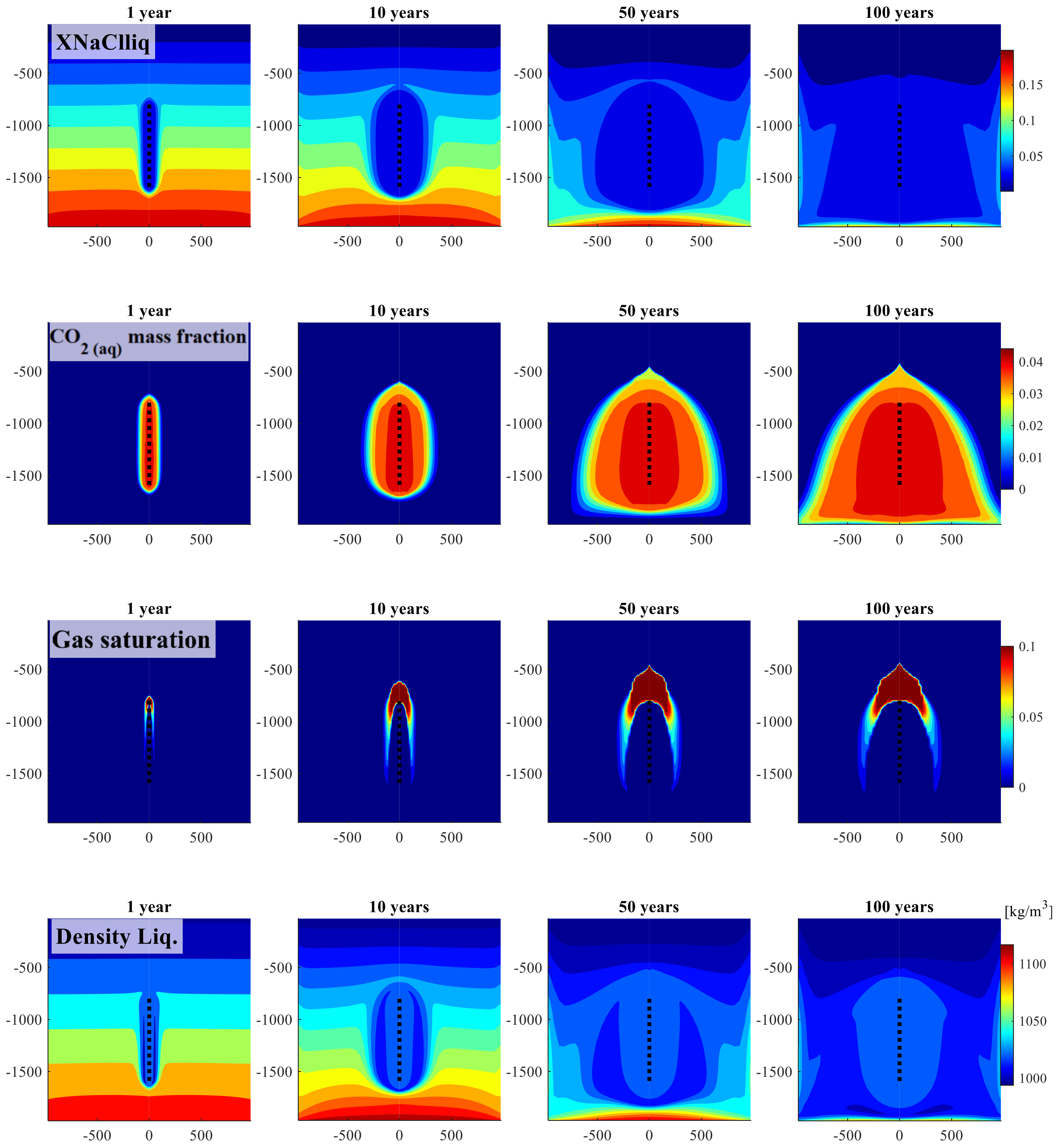

S12. Calculated $CO_2$ concentrations in the subsurface liquid and gas after 10, 50, 100 years of carbonated water injection and 150 years after injection stop. The results shown are for the '100 ppm/m initial salinity gradient system'. This system has a 30 °C/km gradient, a 100 ppm/m initial reservoir salinity, injection water salinity of 35,000 ppm and temperature of 40 °C. The water to $CO_2$ injection mass ratio is 25:1. The upper plots show the 2D cross sections of salinity (NaCl mass fraction in the liquid), followed by the $CO_2$ concentration in the liquid phase, $CO_2$ gas saturation, which is the mass fraction of exsolved $CO_2$ in the pore fluids, whereas the lower plots show 2D cross sections of the density of the liquid phase at the indicated times. Color bars defining the range for each of the properties are displayed on right side. The dotted line marks the injection zone.

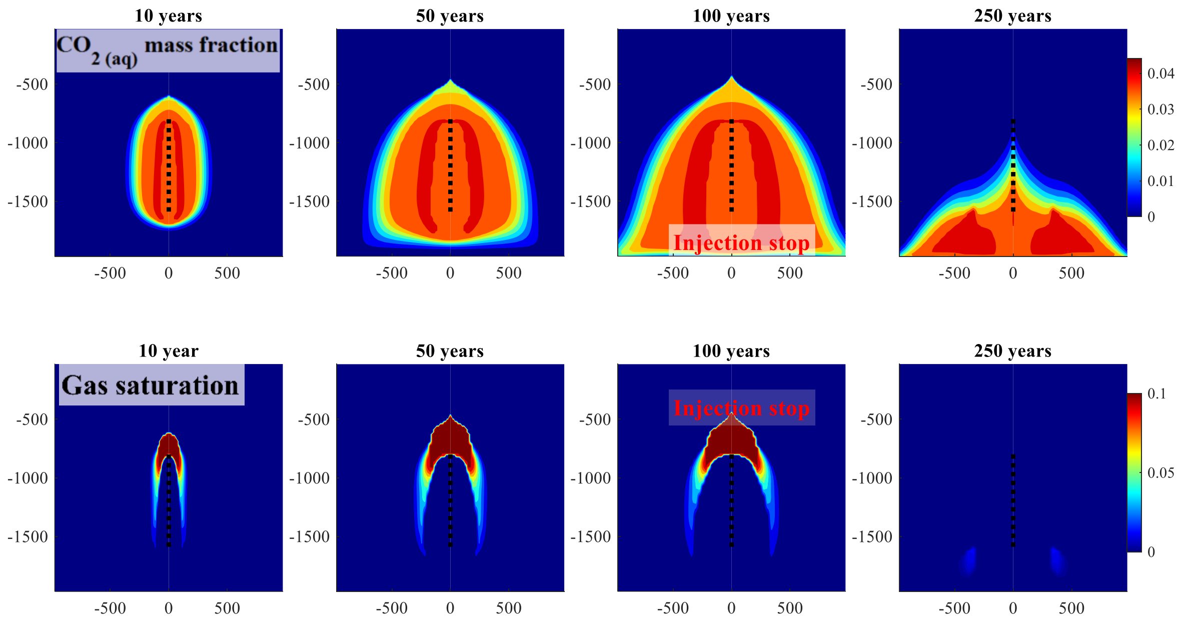

S13. Calculated $CO_2$ concentrations in the subsurface liquid and gas after 1, 10, 50, and 100 years of carbonated water injection. The results shown are for the ‘400 m injection zone thickness system’. This system has a 30 °C/km gradient, a constant salinity of 35,000 ppm, equal to that of seawater, and water injection temperature of 40 °C. The water to $CO_2$ injection mass ratio is 26:1. The upper plots show the 2D cross sections of pressure, followed by the $CO_2$ concentration in the liquid phase, $CO_2$ gas saturation, which is the mass fraction of exsolved $CO_2$ in the pore fluids, whereas the lower plots show 2D cross sections of the $CO_2$ gas saturation, which is the mass fraction of exsolved $CO_2$ in the pore fluids, at the indicated times. Color bars defining the range for each of the properties are displayed on right side. The dotted line marks the injection zone with decreased thickness in this example compared to the reference system.

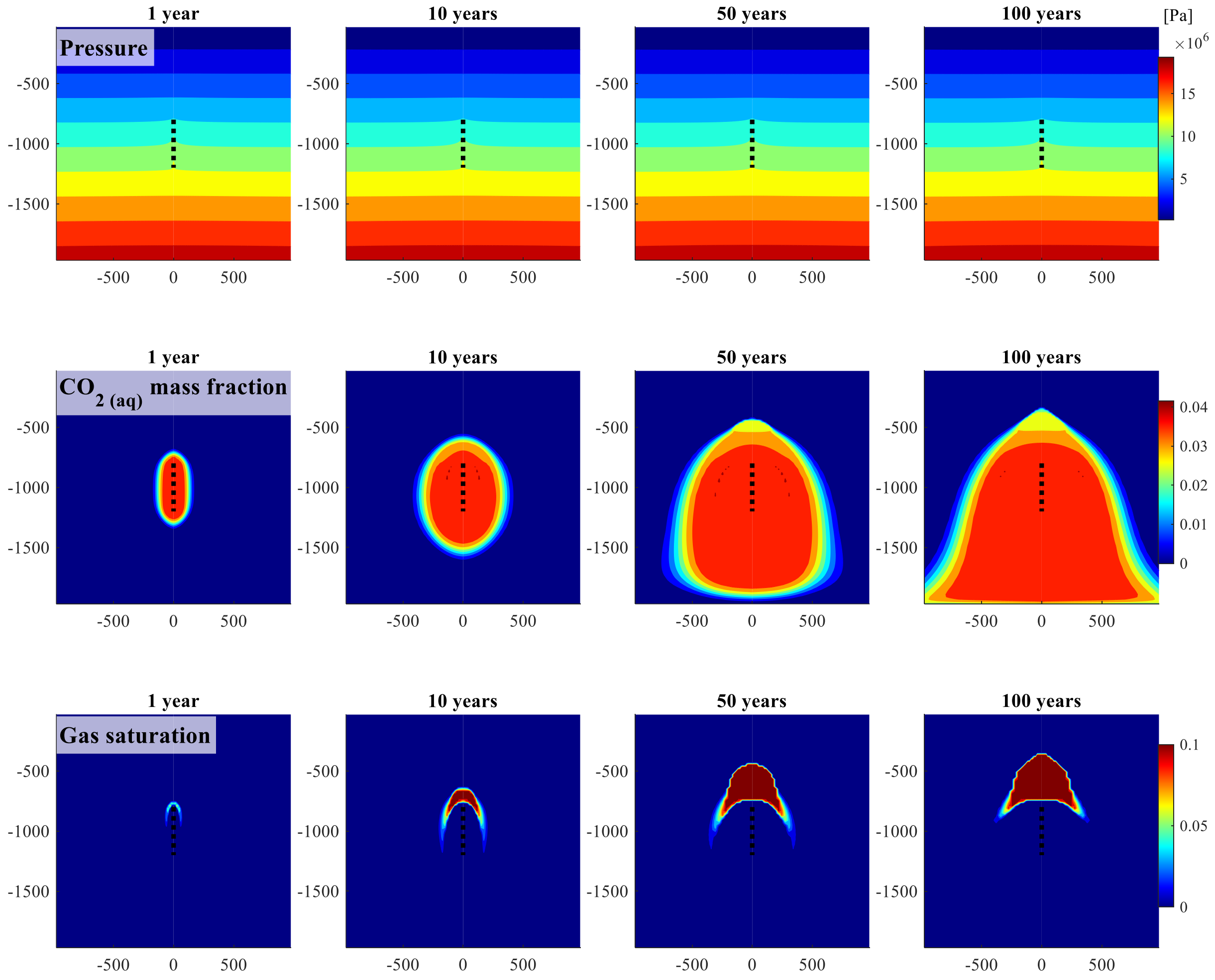

S14. Calculated $CO_2$ concentrations in the subsurface liquid and gas after 1, 10, 50, and 100 years of carbonated water injection. The results shown are for the ‘1200 m injection zone thickness system. This system has a 30 °C/km gradient, a constant salinity of 35,000 ppm, equal to that of seawater, and water injection temperature of 40 °C. The water to $CO_2$ injection mass ratio is 25:1. The upper plots show the 2D cross sections of pressure, followed by the $CO_2$ concentration in the liquid phase, $CO_2$ gas saturation, which is the mass fraction of exsolved $CO_2$ in the pore fluids, whereas the lower plots show 2D cross sections of the $CO_2$ gas saturation, which is the mass fraction of exsolved $CO_2$ in the pore fluids, at the indicated times. Color bars defining the range for each of the properties are displayed on right side. The dotted line marks the injection zone with increased thickness in this example compared to the reference system.

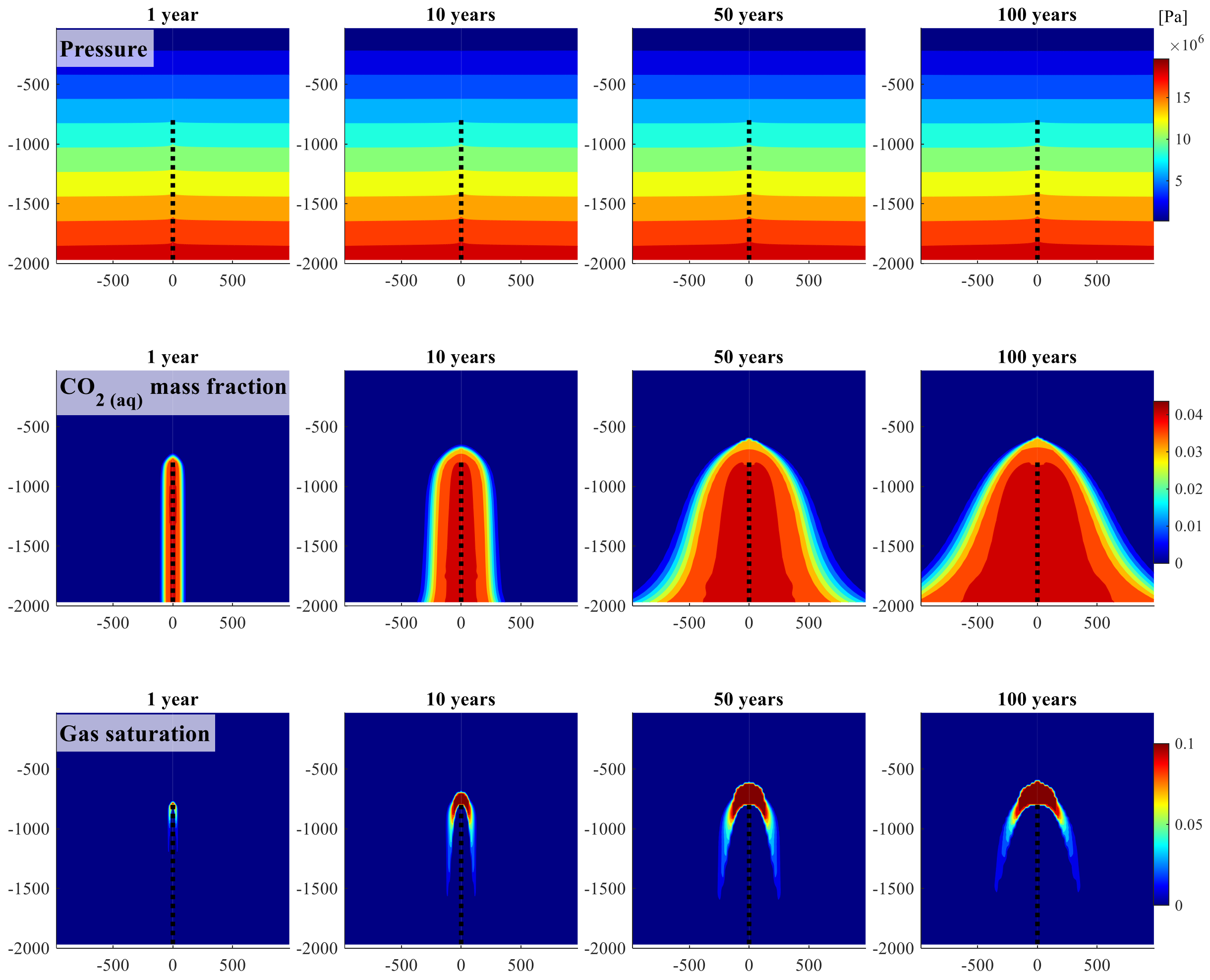

S15. Calculated $CO_2$ concentrations in the subsurface liquid and gas after 1, 10, 50, and 100 years of carbonated water injection. The results shown are for the '1000 to1800 m injection depth system (with an 800 m thick injection zone)'. This system has a 30 °C/km gradient, a constant salinity of 35,000 ppm, equal to that of seawater, and water injection temperature of 40 °C. The water to $CO_2$ injection mass ratio is 23:1. The upper plots show the 2D cross sections of pressure, followed by the $CO_2$ concentration in the liquid phase, $CO_2$ gas saturation, which is the mass fraction of exsolved $CO_2$ in the pore fluids, whereas the lower plots show 2D cross sections of the $CO_2$ gas saturation, which is the mass fraction of exsolved $CO_2$ in the pore fluids, at the indicated times. Color bars defining the range for each of the properties are displayed on right side. The dotted line marks the injection zone.

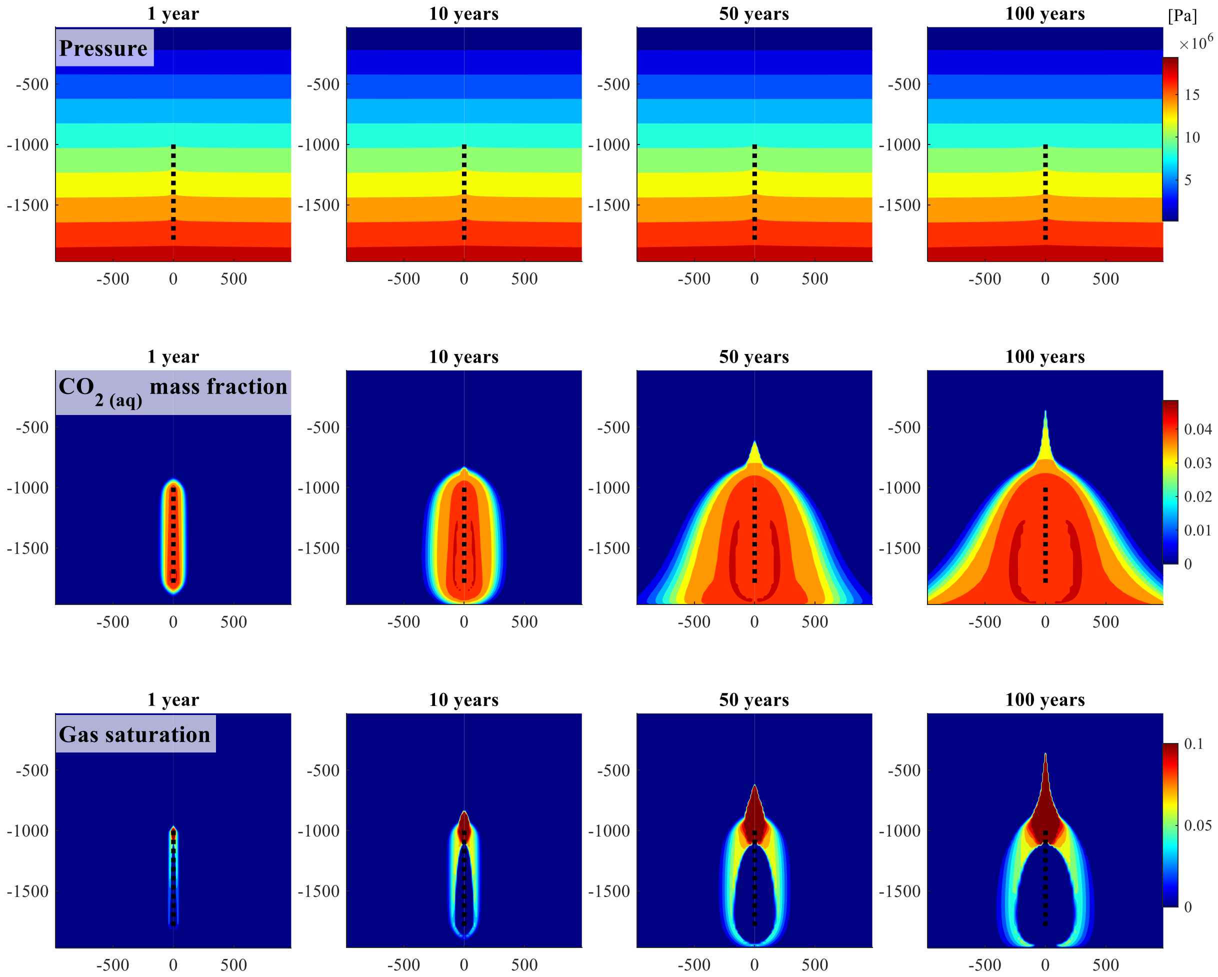

S16. Calculated $CO_2$ concentrations in the subsurface liquid and gas after 1, 10, 50, and 100 years of carbonated water injection. The results shown are for the '1700 to 2000 m injection depth system (with a 300 m thick injection zone)'. This system has a 30 °C/km gradient, a constant salinity of 35,000 ppm, equal to that of seawater, and water injection temperature of 40 °C. The water to $CO_2$ injection mass ratio is 19:1. The upper plots show the 2D cross sections of pressure, followed by the $CO_2$ concentration in the liquid phase, $CO_2$ gas saturation, which is the mass fraction of exsolved $CO_2$ in the pore fluids, whereas the lower plots show 2D cross sections of the $CO_2$ gas saturation, which is the mass fraction of exsolved $CO_2$ in the pore fluids, at the indicated times. Color bars defining the range for each of the properties are displayed on right side. The dotted line marks the injection zone with decreased thickness in this example compared to the reference case.

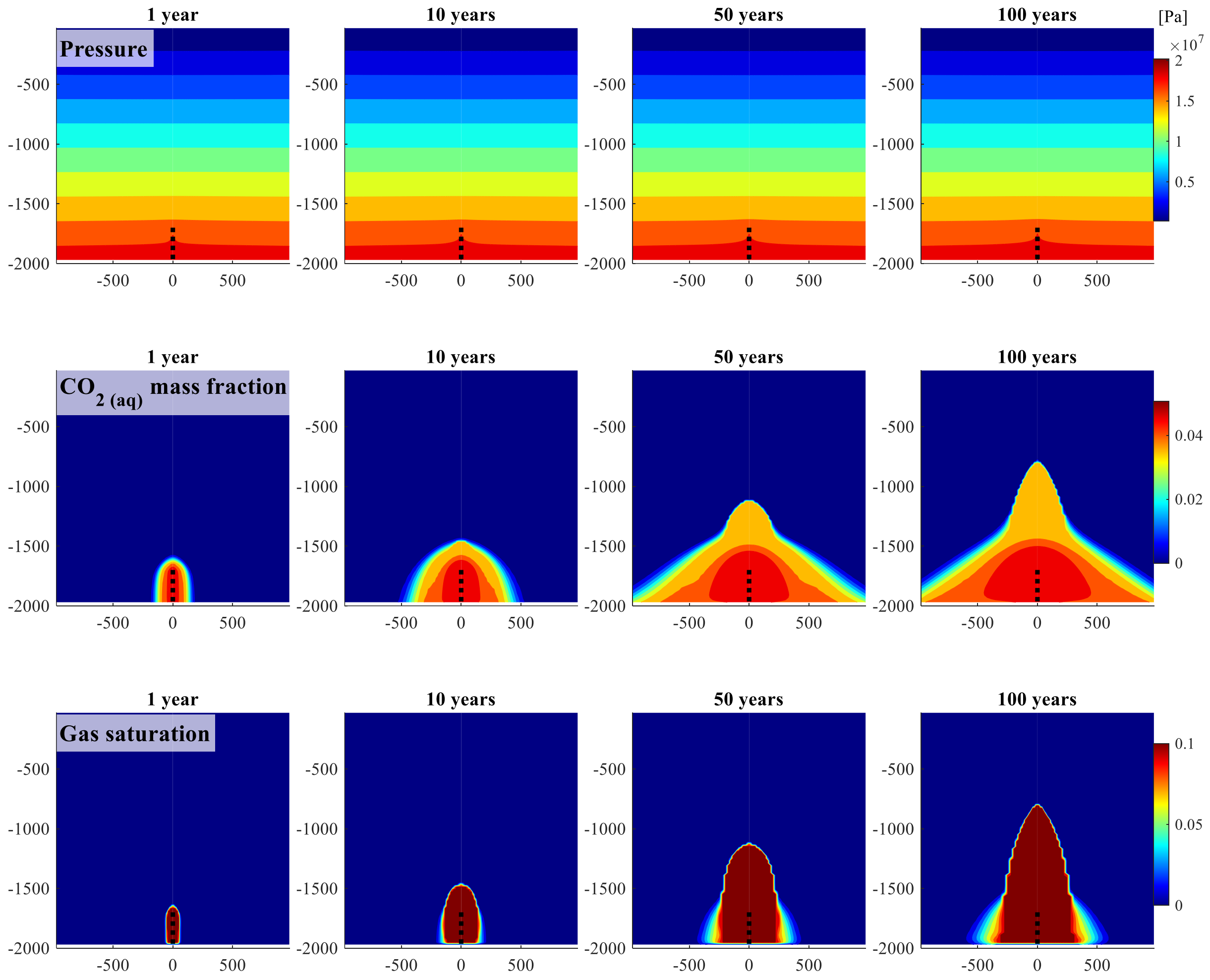

Supplement: Supplementary file 1 — Supplementary Figures. [file 41598_2022_24623_MOESM1_ESM.pdf]
